# Patient-derived xenograft: a developing tool for screening biomarkers and potential therapeutic targets for human esophageal cancers

**DOI:** 10.18632/aging.202934

**Published:** 2021-04-26

**Authors:** Tianfeng Lan, Xia Xue, Louisa Chard Dunmall, Jinxin Miao, Yaohe Wang

**Affiliations:** 1 Sino-British Research Center for Molecular Oncology, National Center for the International Research in Cell and Gene Therapy, School of Basic Sciences, Academy of Medical Sciences, Zhengzhou University, Zhengzhou, Henan, P.R. China; 2 The Academy of Medical Science, Precision Medicine Center of the Second Affiliated Hospital of Zhengzhou University, Zhengzhou University, Henan, P.R. China; 3 Centre for Cancer Biomarkers and Biotherapeuitcs, Barts Cancer Institute, Queen Mary University of London, London, UK; 4 Academy of Chinese Medicine Science, Henan University of Chinese Medicine, Zhengzhou, Henan, P.R. China

**Keywords:** patient-derived xenograft, esophageal cancer, biomarkers, therapeutic targets, immunodeficient mice

## Abstract

Esophageal cancer (EC) represents a human malignancy, diagnosed often at the advanced stage of cancer and resulting in high morbidity and mortality. The development of precision medicine allows for the identification of more personalized therapeutic strategies to improve cancer treatment. By implanting primary cancer tissues into immunodeficient mice for expansion, patient-derived xenograft (PDX) models largely maintain similar histological and genetic representations naturally found in patients’ tumor cells. PDX models of EC (EC-PDX) provide fine platforms to investigate the tumor microenvironment, tumor genomic heterogeneity, and tumor response to chemoradiotherapy, which are necessary for new drug discovery to combat EC in addition to optimization of current therapeutic strategies for EC. In this review, we summarize the methods used for establishing EC-PDX models and investigate the utilities of EC-PDX in screening predictive biomarkers and potential therapeutic targets. The challenge of this promising research tool is also discussed.

## INTRODUCTION

Esophageal cancer (EC) is an aggressive and invasive disease and early diagnosis is clinically challenging. It is associated with one of the highest mortality rates (500,000 per year) and incidence rates (570,000 new cases per year) [1] and the global incidence and mortality of EC are predicted to increase in the coming decades [1, 2]. The growing risk from this malignancy presents a heavy burden on health care providers in almost every population, particularly in Eastern Asia, the world leader in tobacco use, which is one of the most important risk factors for EC [2].

EC can be divided into esophageal squamous cell carcinoma (ESCC) and esophageal adenocarcinoma (EAC) based on the different cell origins. ESCC originates from squamous cells, while EAC originates predominantly from Barrett mucosa [3]. It is known that the incidences of ESCC and EAC vary geographically. ESCC is predominant in East Asia and parts of Africa and accounts for 90% of the new cases of EC every year [4]. The major causes of ESCC include smoking and excessive drinking. Other risk factors are dietary deficiencies, hot beverage intake, achalasia, history of head and neck squamous cell cancer, and radiation therapy [5, 6]. EAC is found more frequently in Europe and North America and is related to chronic inflammation, intestinal metaplasia (Barrett’s esophagus) in the distal esophageal epithelium and obesity [7–9]. Notably, compared to ESCC, the incidence of EAC has increased persistently in some developed countries in recent years [10]. Although age has not been listed as a risk factor of EC, age may affect patient survival and treatment methods [11, 12]. One study demonstrated that overall survival of patients ≥70 years old was shorter, while length of stay was longer than those <70 years old [13]. In another study, patients ≥70 years old were less likely to be subjected to surgery or/and radiotherapy [14]. Given the great challenge of this disease, it is urgently needed to develop more powerful disease models for a better understanding of the pathogenesis of esophageal cancer and developing new approaches to esophageal cancer prevention, early diagnosis, and treatment.

Patient-derived xenograft (PDX) models are established by the engrafting patients’ tumor tissues into immunodeficient mice to obtain a framework that faithfully simulates human cancer biology *in vivo*. Particularly, PDX models largely recapitulate the genetic, phenotypic, and functional characteristics of the primary tumors after transplantation. Here we summarize the methods of PDX model construction for EC and elucidate the practical value of the PDX model in EC treatment, including its use in screening predictive markers and therapeutic targets. PDX models are of great value in understanding cancer progression of EC and developing precision medicine methods to combat EC.

## Methods for establishing EC PDX models and characteristics of EC-PDX

### The engraftment rates of PDX

The engraftment rates of subcutaneous PDX for EC vary from 13.3% to 55.5%, as reported in several studies listed in Table 1. In Table 2, the success rates of PDX for other tumors including neuroblastoma, osteosarcoma, osteosarcoma and so on are listed, which vary from 24% to 100%. The take rate of orthotopic PDX for EC is claimed as 100%, however, the extremely limited sample size (only one case) used in this study may strongly affect the estimation of the take rate [15]. Because of the anatomical location of the esophagus, the establishment of orthotopic model of EC requires advanced surgical techniques, and hardly achieves simplicity or reproducibility [16, 17]. Therefore, orthotopic PDX models for EC are rarely employed. Both resected tumor tissues and endoscopic biopsies are suitable for the engraftment. Resected tumor tissues are often obtained during surgery, while biopsy specimens are obtained during endoscopic examination for pathological confirmation. Tumor tissues or biopsy specimens are then fragmented and these tissue fragments will be directly implanted or blended with Matrigel before implanting into immunocompromised mice for tumor growth and expansion. Esophageal tumor cell populations isolated from ESCC tissues have also been implanted to establish PDX model [18].

**Table 1:** A summary of PDX models for esophageal cancer.

**Histology**	**Tissue type**	**Implantation method**	**Mouse strain**	**Xenograft success rate (%)**	**Refs.**
ESCC	Resected	SC	SCID mice	37/96 (38.5)	[19]
ESCC/EAC	Resected	SC	NSG mice	ESCC 4/12 (25) EAC 13/49 (33)	[20]
ESCC	Resected	SC	SCID mice	14/26 (53.6)	[21]
ESCC	Biopsy	SC	NOD-SCID mice	25/188 (13.3)	[22]
ESCC / EAC	Biopsy	SC	NOD-SCID/NSG mice	ESCC 5/16(31) EAC 8/54(33)	[27]
GEJ adenocarcinoma	Biopsy	Ort	SCID mice	1/1 (100)	[15]
GEJ/ESCC/EAC	Resected/biopsy	IM/SC	SCID/NOD-SCID/NSG mice	IM 13/18 (72) SC 1/6 (16)	[33]
ESCC	Resected	SC	Athymic nude mice	61/110 (55.5)	[36]
ESCC	Resected	SC	NOD-SCID mice	23/54 (42.6)	[42]
GEJ/ ESCC/EAC	Resected	SC	NOD-SCID mice	21/55 (38)	[43]
ESCC	Resected	SC	SICD mice	25/54(46.3)	[39]

ESCC: esophageal squamous cell carcinoma; GEJ: gastroesophageal junction; EAC: esophageal adenocarcinoma; SC: Subcutaneous; Ort: orthotopic; IM: Intramuscular; SCID: C.B17-Prkdc^scid^; NOD-SCID: NOD.C.B17-Prkdc^scid^; NSG: NOD.Cg-Prkdc^scid^Il2rg^tm1Wjl^.

**Table 2:** The success rate of PDX for other tumor types.

**Tumor types**	**Methods**	**Recipient**	**Success rates%**	**Refs.**
neuroblastoma	Ort	NSG/ athymic nude mice	24	[44]
osteosarcoma	Ort	NSG/ athymic nude mice	48	[44]
rhabdomyosarcoma	Ort	NSG/ athymic nude mice	65	[44]
retinoblastoma	Ort	SCID/athymic nude mice	70	[44]
Wilms tumour	Ort	NSG/ athymic nude mice	78	[44]
desmoplastic small round-cell tumour	Ort	NSG/ athymic nude mice	22	[44]
Ewing sarcoma	Ort	NSG/ athymic nude mice	29	[44]
high-grade sarcoma	Ort	NSG/ athymic nude mice	83	[44]
Colorectal cancer	SC	athymic nude mice	52	[45]
Prostate cancer	SC	SCID/NSG/ C57BL/6 pfp/rag2 mice	100-66	[46]

SC: Subcutaneous; Ort: orthotopic; SCID: C.B17-Prkdc^scid^; NOD-SCID: NOD.C.B17-Prkdc^scid^; NSG: NOD.Cg-Prkdc^scid^Il2rg^tm1Wjl^.

Tumor tissues or biopsy specimens from EC patients are termed P0 (Passage zero); established PDX models are termed passage 1 (P1); when P1 tumors reached 500~1500 mm^3^, fresh tumor fragments are harvested from mice and then subsequently re-implanted into other mice (P2, P3, and so on) [19–22]. In general, PDX models undergoing more than 3 passages (P3) are applicable for drug test experiments. If tumor growth was not detected for at least 6 months or the mass was caused by non-epithelial cell proliferation, the engraftment would be considered as a failure.

### The recipients of PDXs

Immunodeficient mice suitable to receive human tumor tissues include athymic nude mice, C.B17-Prkdc^scid^ (SCID) mice, non-obese diabetic.C.B17-Prkdc^scid^ (NOD-SCID) mice, NOD.Cg-Prkdc^scid^Il2rg^tm1Wjl^ (NSG) mice and NODShi.Cg-Prkdc^scid^ Il2rg^tm1Sug^ (NOG/SCID) (Table 1). A spontaneous mutation of *Foxn1* gene in athymic nude mice results in the deteriorated or absent thymus [23]. They are also characterized by the defective differentiation and proliferation of thymic epithelial cells (TECs) and progenitors of T-lymphocytes [23]. However, an intact innate immune system remains and NK cell activity is high, thus engraftment is limited for most primary solid human tumors and impossible for human normal or malignant hematopoietic cells [24]. SCID mice lack both functional T and B lymphocytes because of a *Prkdc* gene deficiency. The concept of SCID now expands to all severely immunodeficient strains of mice, such as those with Recombination activating gene-1/2 mutation (Rag-1^null^/Rag-2^null^). The engraftment takes rates of human tumor cells (including neuroblastoma, colon cancer, and breast cancer cell line) are higher in SCID mice than nude mice [25]. However, moderate NK cell activity remaining in SCID mice restricts the growth of human hematopoietic cells and PDX tumors after implantation. NOD-SCID mice are cultivated by crossbreeding NOD mice and SCID mice. These immunocompromised mice display defective innate immunity, including the dampened activity of NK cell and macrophages, abnormal dendritic cell development and function, and a lack of complement activation [26]. Therefore, NOD-SICD mice are more suitable for the engraftment of human solid tumors and hematopoietic cells that fail in SCID mice. IL-2 receptor subunit gamma (IL-2Rγ) is indispensable for high-affinity signaling for the IL-2, IL-4, IL-7, IL-9, IL-15, and IL-21 receptors. A lack of IL-2Rγ cripples both the adaptive and innate immune system. NSG mice combine the characters of NOD-SICD mice and IL-2Rγ^null^ mice, and are highly receptive to engraftment of human primary tumors. Nevertheless, no significant improvement in primary EC engraftment has been found using NSG mice compared with NOD-SCID mice [27]. Similarly to NSG mice, NOG mice also lack T and B lymphocytes and NK cells and are compatible with human cells and tissues [28]. The engraftment rate of human hematopoietic cells in NOG mice are significantly elevated when compared with NOD-SCID mice [29]. However, there is no evidence indicating NOG mice are superior recipients for EC-PDX.

Most studies that establish a EC-PDX model used animals aged 6-8 weeks for engraftment of patient-derived xenografts, aging mice might not be suitable for xenografts implantation. The reasons may include: (1) The activity of T cell in athymic nude mice tends to increase with the age. Therefore, engraftment rate of tumor cells or tissues could be enhanced in younger mice (5-10 weeks) (reviewed by Szadvari et al [23]); (2) In some aging mice, such as SCID mice, spontaneous thymic and non-thymic tumors may develop and seriously affect their survival, even they are maintained in an SPF, barrier-protected environment [30]; (3) the life spans of immunodeficient mice vary across different species. The median life span of NOD-SCID mice has been reported as 37 weeks, while that of NSG mice was 89 weeks (range, 59–95 weeks) [26, 31]. (4) Inflammatory conditions are also present in aging NSG female mice and contribute to morbidity and mortality in these mice [32].

### The engraftment methods

**Figure 1:**
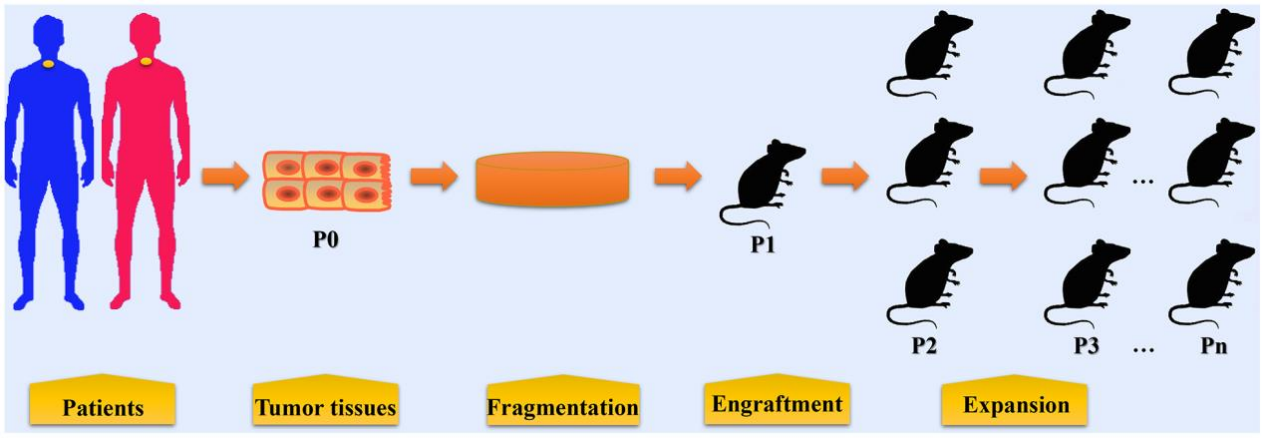
**The procedures in establishing patient-derived xenograft models of esophageal cancer.** Tumor tissues or biopsy are obtained from patients with EC during surgery or endoscopic examination. These tumor tissues and biopsy are termed P0 and are then fragmented before implantation. In some condition, cell populations are isolated from tumor tissues for PDX model establishment. Fragmented samples or primary tumor cells are then implanted into immunocompromised mice (termed P1), either subcutaneously or orthotopically. When P1 tumors reached 500~1500 mm3, fresh tumor fragments are harvested from mice and then subsequently re-implanted into other mice for expansion (P2, P3, and so on).

Currently, subcutaneous, orthotopic, and intramuscular implanting are three methods employed by researchers in the establishment of PDX models for EC (Table 1). Subcutaneous engraftment is a well-established technique employed by most researchers in establishing PDX models. Both resected tumor tissues or biopsy derived from human ESCC or EAC could be engrafted subcutaneously into immunodeficient mice. Orthotopic implantation of human primary EC tissues is scarcely reported. Veeranki et al [15] transabdominally implanted a biopsy sample of EAC at the distal esophagus/gastroesophageal junction to mimic tumor growth patterns in patients. The orthotopic mouse model closely mimics tumor growth patterns seen in patients and recapitulated the response to radiation treatment in patients with EAC [15]. A study showed that intramuscular engraftment might improve the success rate of esophageal PDX establishment (intramuscular vs subcutaneous, 72% vs 16%) [33]. They attributed the improvement to a more abundant blood supply in the muscles than cutaneous tissue. This novel method in tumor tissue engraftment may optimize the process of testing therapeutic drugs for EC. However, lymphomatous transformation occurred in some xenografts when using the intramuscular method [33]. Intramuscular engraftment has also been used in establishing xenograft models for canine osteosarcoma and human ovarian tissues [34, 35]. The feasibility of this engraftment approach should be further validated by more studies. The procedures in establishing PDX models of EC are summarized in Figure 1.

### Characteristics of EC-PDX

Distinguishing features of EC-PDX models support them become useful tools in translational cancer research. Firstly, the morphology and histology in EC-PDX remained consistent when compared that of the corresponding primary tumor tissues [22]. Through 3-4 passages, the degree of differentiation in tumor xenografts varied slightly [22]. Importantly, drug sensitivity, including paclitaxel and cisplatin, in PDX models correlates well with the clinical response in corresponding patients [22]. Thus, EC-PDX model provided a realistic model for drug sensitivity selection for EC patients [22, 36, 37]. Secondly, EC-PDXs are able to mimic the current clinical genetic setting of EC, including mutations in PIK3CA, EGFR, K-Ras, B-Raf and HER2 amplification [19]. These models may support further investigation of the effect of driver gene mutation on treatment response. For instance, the efficacy of Trastuzumab has been developed for the treatment of HER2 positive breast cancer [38]. Likewise, Trastuzumab caused tumor regression in HER2 positive EC-PDX models [39]. However, when PIK3CA mutation was present in the models, Trastuzumab lost the ability to suppress tumor growth, which suggest PIK3CA mutation may be a mechanism of Trastuzumab resistance [39]. Clinical response to chemotherapy using 5-FU and cisplatin was also compromised in EC-PDX models with PIK3CA mutation [19]. Additionally, cancer associated fibroblasts (CAFs) constitute the majority of the tumor microenvironment (TME) [8]. CAFs may promote tumor growth through their mechanical contributions to the stroma and cytokines secretion [40]. Unlike in cell line xenograft, patient-derived CAFs are preserved well in PDX models and contribute to the therapy resistance of EAC [41]. Therefore, PDX models are superior in studying the interaction between EC and TME.

## Application of PDX models in screening predictive biomarkers for chemoradiotherapy

Although multidisciplinary approaches have been developed for the treatment of locally advanced EC, only a small percentage (less than 40%) of patients respond well to these treatments [47]. Many nonresponsive patients may suffer severe adverse effects and even lose the option of surgical resection [48]. Therefore, predictive biomarkers are critical in determining whether chemoradiotherapy solutions are suitable and effective in preventing EC progression in patients. Identification of predictive biomarkers would facilitate accurate risk stratification of patients for therapy and avoid potential morbidity due to ineffective treatment. The employment of PDXs in screening biomarkers has been carried out by many researchers. For instance, CAFs derived from EAC PDXs were shown to play important roles in inducing resistance to chemoradiotherapy [41]. Interleukin-6 (IL-6) produced from/by CAFs drives EMT and enhances cell migration and survival in EAC [41]. Therefore, IL-6 expression from CAFs may provide value in prediction of patient resistance to chemotherapy and radiotherapy [41]. TP53-induced glycolysis and apoptosis regulator (TIGAR) is a downstream regulator of p53 and highly expressed in many hematologic and solid tumors, including leukemia, breast cancer, and EC [49, 50]. TIGAR remodels energy metabolism in ESCC cells and promotes cell proliferation and colony formation [51]. Compared to ESCC-PDXs with low TIGAR expression, those with TIGAR overexpression were more resistant to 5-fluorouracil/Cisplatin, whereas they were sensitized by a glutaminase inhibitor, CB-839 [51]. Therefore, TIGAR expression in EC tissues might be a predictive biomarker in guiding chemotherapeutic strategies [51]. Furthermore, NAD(P)H quinone dehydrogenase 1 (NQO1), an enzyme involved in cellular reactive oxygen species clearance [52], showed enhanced expression in ESCC cells during the treatment of a preparation of curcumin (THC) and was associated with THC resistance [53]. However, the combination of THC and NQO1 inhibitor exerted a superior effect on tumor growth than THC monotherapy in ESCC-PDX, suggesting that NQO1 expression might be a critical biomarker of THC response in ESCC patients [53].

## Application of PDX models in evaluating therapeutic targets for chemotherapy

### Topoisomerase I

Topoisomerase I binds to the supercoiled DNA and cleaves the phosphate backbone of the DNA to release supercoiled DNA [54]. It functions as a critical nuclear enzyme that facilitates DNA replication, transcription, recombination and repair [55–57]. High expression of topoisomerase I can be found in human ESCC tissues and is related to poor prognosis, while topoisomerase I expression is relatively low in the normal squamous epithelium [58, 59]. Gimatecan is a modified lipophilic analog of camptothecin [60], which exerts anti-tumor activity through specifically inhibiting topoisomerase I activity. Gimatecan can induce DNA damage, S-phage arrest and apoptosis in ESCC cells in cell-line-derived xenograft (CDX) models as well as in PDX models through suppressing the expression and function of topoisomerase I [61]. (S)-10-Hydroxycamptothecin (HCPT) is another topoisomerase I inhibitor isolated from *Camptotheca cuminata*. HCPT suppresses the enzymatic activity of topoisomerase I, impedes cell proliferation, and induces cell cycle arrest and apoptosis in ESCC cells [59]. The tumor growth of PDX models was also suppressed by HCPT, supporting its anti-tumor activity [59]. Both studies validated the antitumor efficacy of topoisomerase I inhibitors in ESCC cells and PDX models, which may pave the way for the clinical use of these drugs in the treatment of EC.

### EGFR and HER2

The HER family of receptor tyrosine kinases contains epidermal growth factor receptor (EGFR/ErbB1/HER1), erb-b2 receptor tyrosine kinase 2 (ERBB2/HER2/Neu), erb-b2 receptor tyrosine kinase 3 (ERBB3/HER3), and erb-b2 receptor tyrosine kinase 4 (ERBB4/HER4) [62, 63]. The aberrant activation of these receptor tyrosine kinases facilitates the tumorigenesis and progression of multiple malignant tumors, such as EC, lung cancer, gastric cancer, and colon cancer [64]. EGFR and HER2 are overexpressed in human primary EC tissues and significantly associated with overall survival in EC [65, 66]. The effect and mechanism of inhibitors targeting EGFR and HER2 have been evaluated using EC-PDX models. Theliatinib is a potent and highly selective EGFR inhibitor currently in Phase I clinical study in China (NCT02601248). Theliatinib was effective in restraining the tumor growth of ESCC-PDX models with EGFR gene amplification [42]. However, PIK3CA mutation or FGFR1 over-expression in PDX attenuated the effect of theliatinib, suggesting care to apply theliatinib to only responsive subsets of patients is required [42]. Cetuximab is a mouse-human chimeric antibody that functions through binding with EGFR, leading to an inhibition of EGFR phosphorylation and activation [67]. Zhu et al [36] tested the response of ESCC to cetuximab via PDX models. It is notable that EGFR amplification, *EGFR* mRNA levels and EGFR protein expression could be significantly correlated with the ESCC-PDXs response to cetuximab treatment [36].

The anti-HER2 monoclonal antibody, trastuzumab has been shown its efficacy in prolonging overall survival of patients with HER2-positive advanced gastric or gastro-oesophageal junction cancer [68, 69]. Trastuzumab resistance was observed in PDX models of ESCC with a dose-dependent decrease in HER2 expression and a significant increase of HER3 and HER4 expression [70]. The HER3 might be a potential therapeutic targets for trastuzumab resistant cancer, as inhibition of HER3 could reverse trastuzumab resistance in ESCC and EAC cells [70]. Afatinib is a pan-HER inhibitor for clinical treatment of lung cancer and breast cancer [71, 72]. EGFR gene amplification or overexpression was a predictor for afatinib sensitivity of ESCC [73]. Afatinib inhibited the phosphorylation of EGFR, S6, and ERK and induced G1 phase arrest and apoptosis in ESCC cells and PDX models [73]. In the PDX model of esophagogastric cancer, afatinib resistance could be caused by MET amplification, which might be overcome by MET inhibitor [74]. Additionally, lapatinib, a dual tyrosine kinase inhibitor of EGFR2 and HER2, was able to contain tumor growth of PDX in combination with 5-fluorouracil [75].

### Aurora-A and -B

Aurora-A and -B are two members of Aurora family kinases that are implicated in the control of mitosis [76, 77]. Aurora-A is required for centrosome maturation and separation and bipolar spindle assembly, while Aurora-B regulates cytokinesis and acts as a member of the chromosome passenger complex [77]. Both Aurora-A and -B can be found overexpressed in human EC tissues [78, 79] and act to enhance cell invasion and malignant phenotypes in ESCC [80, 81]. Treatment of APIO-EE-9, an Aurora kinase inhibitor, resulted in inhibition of cell growth and proliferation, induction of apoptosis, and reduction of Aurora-A and -B activities in ESCC cell lines [82]. In PDX models of ESCC, APIO-EE-9 effectively inhibited tumor growth with minimal toxicity [82]. The inhibition of Aurora-A and -B might be effective in reducing uncontrolled proliferation in ESCC, thus contributing to tumor suppression.

### Cyclin-dependent kinase 4/6/9

Cyclin-dependent kinases (CDKs) can integrate many extracellular signaling pathways to drive cell cycle transition [83]. The employment of CDK4/6 inhibitors, such as palbociclib, ribociclib, and abemaciclib for clinical treatment of breast cancer has been approved in the United States [84, 85]. These inhibitors may interrupt the hyperactive cyclin D associated kinases in Rb positive tumor cells, resulting in cell cycle arrest [84]. The effectiveness of CDK4/6 inhibitors for EC therapy has been also investigated in preclinical and clinical trials [86–88]. The results of high-throughput sequencing in EACs showed that more than half of EACs contained biomarkers of response to CDK4/6 inhibitors [89]. SHR6390 is an orally bioavailable inhibitor of CDK4/6. Suppression of proliferation of EC cells and tumor growth of PDX model were observed following SHR6390 treatment [90]. The combination of SHR6390 with paclitaxel or cisplatin synergistically inhibited tumor growth in a PDX model [90]. The effects of another two CDK4/6 inhibitors, palbociclib (PD-0332991) and ribociclib (LEE011) on human ESCC were validated *in vitro* and using CDX and PDX models [37]. ESCC cells and PDX models with cyclin dependent kinase inhibitor 2A (CDKN2A) or CDKN2B loss were more sensitive to palbociclib and ribociclib treatment than cells with wild-type genes [37]. Intriguingly, through using a mouse avatar model of ESCC, the authors demonstrated that CDKN2A and CDKN2B loss were critical biomarkers for CDK4/6 inhibitor therapy [37]. Lastly, researchers examined the expression of CDK9 in human EAC tissues and Barrett's esophagus and found that CDK9 was overexpressed in EAC [91]. Pharmaceutical inhibition of CDK9 by Flavopiridol and CAN508 diminished cell proliferation and promoted apoptosis in EAC cells [91]. Furthermore, the treatment using a CDK9 inhibitor might enhance the cell-killing effect of radiation on EAC. Synergetic effect of the CDK9 inhibitor, BAY1143572 and radiation were assessed in EAC cell lines and PDX models [92]. By inhibiting CDK9 activation, BAY1143572 could sensitize EAC cells and PDX of EAC to radiation [92]. The precise mechanism by which CDK4/6/9 inhibitors suppress EC progression remains unclear and needs further investigation.

### JAK/STAT3 signaling pathway

Janus kinase (JAK)/ signal transducer and activator of transcription 3 (STAT3) signaling activation frequently present in primary ESCC and is associated with poor prognosis in patients [93]. JAK/STAT3 can be recruited by EGFR and contributes to esophageal keratinocyte migration [94]. Aberrant activated STAT3 in cancer cells induces epithelial mesenchymal transition and facilitated metastasis [95]. Suppressor of cytokine signaling 1 (SOCS1) is a multifunction protein that functions as a signal inhibitor and a regulator in the process of ubiquitination [96]. SOCS1 negatively regulates JAK/STAT3 signaling transduction via interaction with JAK proteins [97]. Overexpression of SOCS1 using recombinant adenoviral vectors reduced cell proliferation and inactivated JAK/STAT3 signaling in ESCC cells as well as in ESCC PDX models [98]. By restraining the JAK/STAT3/c-MYC pathway, metformin could inhibit the transition of normal endothelial cells toward tumor endothelial cells induced by tumor conditioned medium [99]. In the human ESCC PDX model, metformin prevented tumor growth and tumor angiogenesis [99]. The phosphorylation of STAT3 in ESCC cells could be blocked by a small molecular STAT3 inhibitor, Stattic [100]. Stattic alone or in combination with 5-fluorouracil markedly suppressed tumor growth of ESCC-PDX, with less cell proliferation and increased apoptosis in xenografts [100].

### The MAPK cascades

Mitogen-activated protein kinase kinase (MEK)/ extracellular signal-regulated kinase (ERK) signaling is an essential component of the mitogen-activated protein kinase (MAPK) cascades [101]. Mutations of MEK/ERK signaling are frequently seen in many human tumors, including EC [102], lung cancer [103], and breast cancer [104]. Researchers have taken efforts to develop inhibitors of MEK and ERK as cancer therapeutic agents [105, 106]. Purpurogallin, a phenol distilled from oak nutgalls, inhibits the function of MEK1 and MEK2 by binding within their ATP-binding pocket [107]. Purpurogallin inhibited the malignant phenotypes of ESCC cells and tumor growth of ESCC PDX model by targeting MEK1 and MEK2 [107]. Ethyl gallate (EG) is a natural phenolic compound obtained from herbs like *Galla Rhois, Longan* and *Acacia nilotica Wild* [108–110]. EG directly interacts with ERK1/2 and negatively regulated ERK1/2 activities in ESCC cells, leading to the inhibition of cell proliferation, interruption of cell cycle, and increase of cell apoptosis [111]. In ESCC PDX models, EG administration suppressed tumor growth via the inactivation of ERK1/2 [111]. MSK2 acts as a downstream of the ERK1/2 or p38 MAPK pathways and has a regulatory effect on CREB and histone H3 [112, 113]. MSK2 activation as well as downstream CREB-Bcl-2 pathway could be dampened by sulforaphene, leading to the induction of apoptosis and cell cycle arrest and inhibition of cell migration and invasion in EC cells [114] and using EC-PDX models, the anti-tumor effect of sulforaphene was validated [114]. Finally, MKK3/6 acts as an upstream activator of p38 MAPK. A hexahydroxylated flavonoid named gossypetin reduces cell viability and anchorage-independent growth and induces apoptosis in ESCC through binding with MKK3 and MKK6 [115]. Using an ESCC PDX model, the anti-tumor activity of gossypetin was further demonstrated *in vivo* [115].

### Glypican-1

Glypican-1 is a cell surface proteoglycan that presents in a variety of solid tumors and modulates tumor growth, invasion and progression [116]. Glypican-1 overexpression is associated with cisplatin resistance and promotes malignant transition of ESCC via the PTEN/AKT/β-catenin signaling pathway [117, 118]. Glypican-1 expression is relatively weaker in human normal heart, kidney, small intestine, colon and esophageal tissues compared to ESCC tissues [119]. Knockdown of glypican-1 inhibits cell growth and the activation of EGFR, AKT and p44/42-MAPK signaling pathways [119]. Targeting glypican-1 using anti-glypican-1 monoclonal antibody restrained tumor growth and promoted apoptosis in ESCC PDX models [119].

### Hedgehog signaling

Hedgehog signaling pathway is critical for tissue development, injury repair and tumorigenesis [120, 121]. The Hedgehog signaling cascade contains 3 ligands, Sonic (SHH), Indian, and Desert Hedgehog, which activate downstream signal transducer protein smoothened (SMO) and subsequently the GLI protein family (GLI1, GLI2, and GLI3) by binding with the transmembrane receptor Patched-1 [122]. Aberrant activation of Hedgehog signaling is linked to cancer progression and chemoresistance. GLI1 activity was elevated in EAC and correlated with EAC differentiation as well as the response to neoadjuvant chemotherapy [123]. In the established EAC PDX models, upregulation of hedgehog ligands (e.g. SHH) was found in tumor epithelium and upregulation was further enhanced by radiation treatment [20, 124]. Unlike EAC and Barrett’s Esophagus, SHH expression in ESCC is relatively rare [125]. Hence, researchers concentrated on developing inhibitors targeting SHH signaling for invasive EAC [126, 127]. Evidence has shown that the Hedgehog signaling pathway is a target for improving chemoradiation therapy in EC [128]. SHH inhibition using a monoclonal antibody 5E1 augmented the growth delay of PDX tumors following radiation [124]. Likewise, SMO inhibition with an SMO inhibitor, LDE225, also increased growth delay induced by radiation [124].

### PI3K/AKT signaling pathway

The phosphatidylinositol-4,5-bisphosphate 3-kinase (PI3K)/ serine/threonine kinase 1 (AKT) signaling pathway plays a critical role in modulating cellular processes such as cell proliferation, survival, protein synthesis and glucose homeostasis [129, 130]. The PI3K/AKT signaling pathway can be activated by different receptor tyrosine kinases (RTKs) including the EGFR family, insulin-like growth factor 1 (IGF-1) receptor, and fibroblastic growth factor [131]. The strategies that target the PI3K/AKT pathway help to inhibit cadherin switching, diminish cell proliferation and migration, alleviate inflammation, restore chemosensitivity, and increase radiosensitivity in EC cells. For instance, the combination of a clinical PI3Kα-selective inhibitor CYH33 and radiation promoted DNA damage, cell cycle arrest and apoptosis in ESCC cells [132]. In the PDX model, CYH33 and radiation inhibited tumor growth, lowered Akt phosphorylation and M2-like macrophage infiltration [132]. Oridonin, Xanthohumol, and Scutellarin are natural compounds isolated from herbs. Their activities in targeting AKT activation in ESCC cells and ESCC-PDX have been reported [133–135]. These AKT inhibitors were effective in suppressing cell growth and inducing cell cycle arrest in ESCC cells as well as decreasing PDX tumor growth *in vivo*. Importantly, the effects of these inhibitors were dependent on AKT protein level in ESCC cells [134, 135].

### VEGFR2

The vascular endothelial growth factor (VEGF)/vascular endothelial growth factor receptor 2 (VEGFR2) system plays an important role in tumor angiogenesis. Patients with solid tumors have demonstrated benefit from drugs targeting VEGF and/or VEGFR2 [136, 137]. Ramucirumab is an anti-VEGFR2 monoclonal antibody that may prevent VEGFR2 dimerization and thus suppress downstream signaling transduction [138]. VEGFR2 expression was found to be significantly elevated in EC tissues and correlated with poor efficacy of cytotoxic treatment [139]. Ramucirumab has been approved by FDA for treating gastric and GEJ adenocarcinomas either as a single agent or in combination with paclitaxel [140, 141]. Apatinib, a broad inhibitor of VEGFR2, RET, c-Kit and c-Src, induced cell apoptosis and cell cycle arrest, inhibited malignant transformation and sensitized EC to cisplatin [139]. The efficacy of apatinib monotherapy as second- or further-line treatment for advanced EC has been validated in a Phase II study [142]. Apatinib also exhibited its potential efficacy in patients with metastatic ESCC when combined with docetaxel [143]. Moreover, inhibition of VEGFR2 using DC101, a murine VEGFR2 inhibitor, delayed tumor growth and prolonged survival of animals with EAC xenografts [144]. However, vascular regression induced by DC101 impaired the uptake of intraperitoneally administered nab-paclitaxel [144]. This study suggested the limits of the combination of anti-angiogenesis and cytotoxic agents in EAC therapy [144].

### HSP90

A significant correlation between heat shock protein 90 (HSP90) expression and Her2 status has been found in EAC [145]. Serum HSP90a level was a significant predictor for definitive chemoradiotherapy in patients with ESCC [146]. The reduction ratio of HSSP90a could be an independent prognostic factor for ESCC patients [146]. The detailed role of HSP90 as a therapeutic target in EC has been reviewed in a previous study [147]. Drugs targeting HSP90 alone or combined with other chemotherapeutic drugs (i.e. cisplatin) and radiation play inhibitory roles in EC cell survival. For instance, the inhibitor of HSP90 Ganetespib (STA-9090) could inhibit cell proliferation and induce apoptosis in ESCC cells and PDX models [148]. Interestingly, the effect of HSP90 inhibition seemed to be dependent on MYC expression. ESCC cells and xenografted primary tumors overexpressing MYC were more sensitive to STA-9090 [148].

### Notch signaling pathway

Dysregulation of notch signaling due to NOTCH1, NOTCH2 or NOTCH3 gene mutation has been shown in ESCC [149, 150]. Nuclear accumulation of notch intracellular domain (NICD) is closely linked to tumor grade and stage in human ESCC [151]. Higher expression of NICD is detected in human EAC tissues compared with the normal esophageal mucosa and the normal gastric cardia [152] and NICD expression correlates with the stage of EAC. Notch signaling regulates EAC cell proliferation and transformation of normal esophageal epithelial cells [152, 153]. DAPT treatment suppressed tumor growth and promoted apoptosis in EAC CDX models and PDX models [152]. Inhibition of Notch signaling also decreased the expression of cancer stem-cell markers in EAC cells [152].

### Other targets and utilities

Microtubules are composed of alpha- and beta-tubulin heterodimers, the basic structures that are essential for cell shape and behavior. Microtubules are highly dynamic structures that change during the cell cycle. Clinically, tubulin binding agents (TBA) can suppress microtubule dynamics and induce cell cycle arrest, thus contributing to tumor growth inhibition [154, 155]. PPMP (2-[4-(3,4-dimethoxyphenyl)-3-methyl-1H-pyrazol-5-yl]-5-[(2-methylprop- 2-en-1-yl)oxy]phenol), a novel TBA, reduced cell viability, caused cell cycle arrest and apoptosis in ESCC cell lines [156]. PPMP might occupy the colchicine binding site of tubulin and inhibit tubulin polymerization in ESCC cells [156]. *In vivo*, PPMP effectively suppressed tumor growth in animals bearing ESCC PDX [156].

With the extensive application of next-generation sequencing to cancer transcriptomes, the role of long non-coding RNAs (LncRNAs) in tumor progression has increasingly drawn people’s attention [157]. LncRNAs act as tumor suppressors or oncogenes by modulating tumor-suppressive or oncogenic pathways [158]. LncRNA AGPG is highly enhanced in human ESCC tissues and cell lines. AGPG interacts with PFKFB3, contributing to metabolism remodeling in ESCC cells. Administration of an AGPG inhibitor to ESCC PDX models markedly reduced tumor growth [159].

Additionally, PDXs are also of great value in establishing chemoresistant cell lines and identity new therapeutic targets. Liu et al [18] established cisplatin-resistant ESCC cell lines through repeatedly treating ESCC-PDX models with cisplatin. With these cisplatin-resistant ESCC cells, they were able to pick out microRNA-455-3p as a potential therapeutic target to overcome drug resistance in EC patients [18].

## CONCLUSIONS

**Figure 2:**
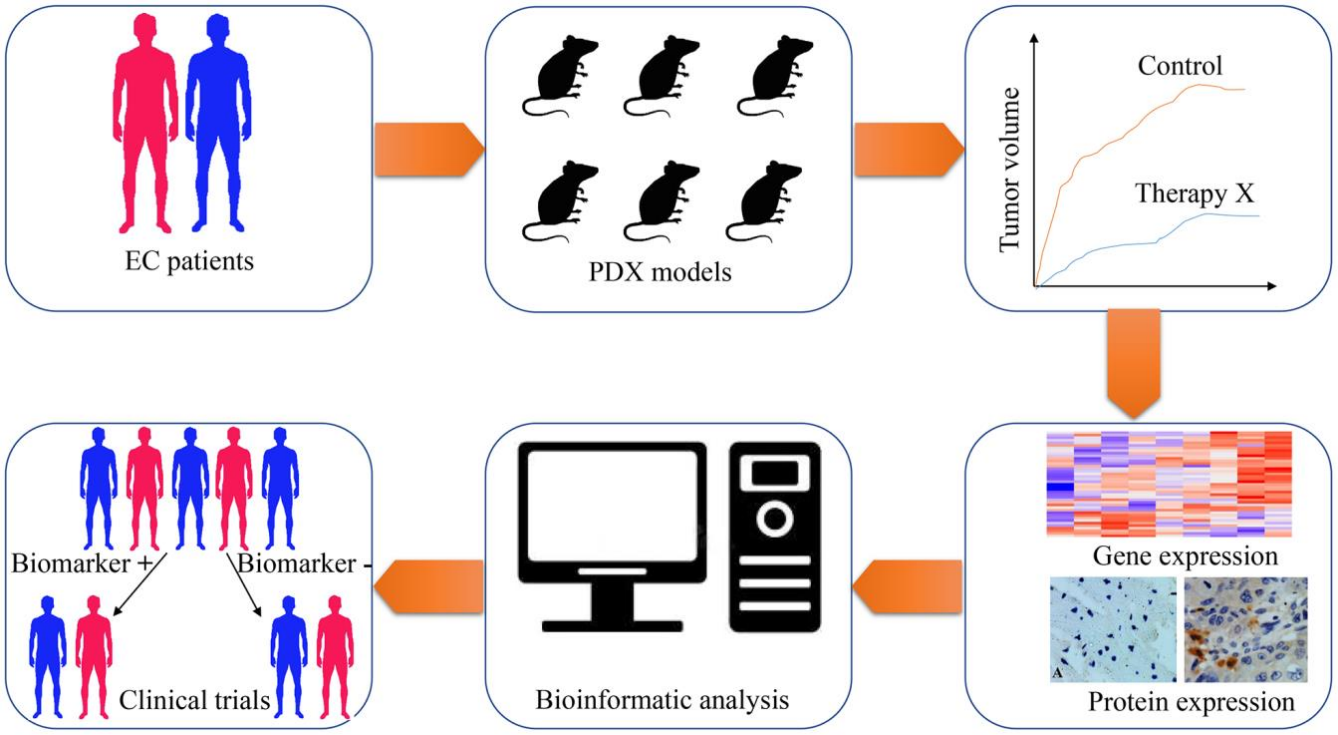
**The application of patient-derived xenograft (PDX) in screening predictive biomarkers and therapeutic targets for esophageal cancer therapy.** Esophageal cancer tissues are obtained from patients and implanted into immunodeficient mice for PDX models establishment. With the PDX models, the treatment response of chemotherapeutic drugs, radiotherapeutic methods or targeted drugs are tested on these tumor xenografts. Subsequently, genome-wide sequencing techniques and expressional analysis are carried out to screen genes with differential expression, which are related to various therapeutic methods. Through bioinformatic analysis, potential biomarkers are selected from differentially expressed genes. Finally, clinical trials are designed and performed to validate the feasibility of these biomarkers.

PDX models of EC are increasingly utilized for studying tumor biology, investigating genetic heterogeneity, and screening predictive biomarkers and therapeutic targets (Table 3). Indeed, investigators have tested various drugs or radiation therapy on mice bearing PDX and screened predictive biomarkers/ therapeutic targets that may guide for EC therapy in patients (Figure 2).

**Table 3:** Agents and their targets tested in PDX models of esophageal cancer.

**Agent**	**Target**	**Histology**	**Administration method**	**Mouse strain**	**Reference**
Gimatecan	Topoisomerase I	ESCC	Oral gavage	NOD-SCID mice	[61]
HCPT	Topoisomerase I	ESCC	Paraneoplastic injection	SCID mice	[59]
Cetuximab	EGFR	ESCC	Intraperitoneal injection	Athymic nude mice	[36]
Theliatinib	EGFR	ESCC	Oral gavage	NOD-SCID mice	[42]
Trastuzumab	HER2	EAC	Intraperitoneal injection	NSG mice	[70]
Trastuzumab/ pertuzumab	HER2/HER3	EAC	Intraperitoneal injection	NSG mice	[160]
Afatinib dasatinib	EGFR /Src family kinase	ESCC	Oral gavage	NOD-SCID mice	[73]
Afatinib/AMG 337	HER2/MET	EG	-	-	[74]
Lapatinib	EGFR/HER2	ESCC	Oral gavage	Athymic nude mice	[75]
APIO-EE-9	Aurora A and B	ESCC	-	SCID mice	[82]
SHR6390	CDK4/6	ESCC	Oral gavage	NOD-SCID mice	[90]
Palbociclib	CDK4/6	ESCC	Oral gavage	BALB/c nude mice	[37]
BAY1143572	CDK9	EAC	Intraperitoneal injection	Athymic nude mice	[92]
AdSOCS1	SOCS1	ESCC	Intratumoral injection	NOD-SCID	[98]
Metformin	JAK/STAT3	ESCC	-	SCID mice	[99]
Stattic	STAT3	ESCC	Intraperitoneal injection	SCID mice	[100]
Purpurogallin	MEK1/2	ESCC	Oral gavage	SCID mice	[107]
Ethyl gallate	ERK1/2	ESCC	Oral gavage	SCID mcie	[111]
Sulforaphene	MSK2	ESCC	Intraperitoneal injection	SCID mice	[114]
Gossypetin	MKK3/6	ESCC	Oral gavage	SCID mice	[115]
Anti-Glypican-1 mAb	Glypican-1	ESCC	Intraperitoneal injection	NOG/SCID mice	[119]
5E1 LDE225	SHH SMO	EAC	Intraperitoneal injection Oral gavage	NOD-SCID/NSG mice	[124]
CYH33	PI3Kα	ESCC	Oral gavage	BALB/c nude mice	[132]
Oridonin	Akt	ESCC	Oral gavage	SCID mice	[133]
Xanthohumol	Akt	ESCC	Oral gavage	SCID mice	[134]
Scutellarin	Akt1/2	ESCC	Oral gavage	SCID mice	[135]
DC101	VEGFR2	EAC	Intraperitoneal injection	Athymic nude mice	[144]
Ganetespib	HSP90	ESCC	Intraperitoneal injection	NSG mice	[148]
DAPT	Notch signaling	EAC	Intraperitoneal injection	NSG	[152]
PPMP	Tubulin	ESCC	Intraperitoneal injection	SCID mice	[156]
Antisense oligonucleotides	LncRNA AGPG	ESCC	Intratumoral injection	Athymic nude mice	[159]

HCPT: (S)-10-Hydroxycamptothecin; EGFR: epidermal growth factor receptor; HER2: erb-b2 receptor tyrosine kinase 2; HER3: erb-b2 receptor tyrosine kinase 3; MET: MET proto-oncogene, receptor tyrosine kinase; CDK4/6/9:cyclin dependent kinase 4/6/9; SOCS1:suppressor of cytokine signaling 1; JAK: Janus kinase; STAT3:signal transducer and activator of transcription 3; MEK1/2:mitogen-activated protein kinase kinase 1/2; ERK1/2: extracellular signal-regulated kinase 1/2; MSK2:ribosomal protein S6 kinase A4; MKK3/6:mitogen-activated protein kinase kinase 3/6; SHH: sonic hedgehog; SMO: smoothened, frizzled class receptor; PI3Kα:phosphatidylinositol-4,5-bisphosphate 3-kinase catalytic subunit alpha; Akt: serine/threonine kinase 1; VEGFR2: vascular endothelial growth factor receptor 2; HSP90: heat shock protein 90; EAC: esophageal adenocarcinoma; ESCC: esophageal squamous cell carcinoma; EG: esophagogastric cancer; SCID: C.B17-Prkdc^scid^; NOD-SCID: NOD.C.B17-Prkdc^scid^; NOG/SCID: NODShi.Cg-Prkdc^scid^ Il2rg^tm1Sug^; NSG: NOD.Cg-Prkdc^scid^Il2rg^tm1Wjl^.

However, there are still several problems that need to be solved in the establishment and usage of EC-PDX:

1. The engraftment rates of EC-PDX remain relatively low with the current methods, and only a minority of tumor tissues derived from patients can be successfully engrafted. As a result, there is a high cost in establishing successful PDX models. To solve this issue, novel immunodeficient animals are needed, such as gene-modified rats and hamsters [161–163];
2. Although subcutaneous engraftment is commonly employed by most researchers [164], subcutaneous models less accurately reflect tumor progression compared with orthotopic methods and hinder the investigation of tumor metastasis, angiogenesis and tumor microenvironment in EC. The difficulties in establishing and examining orthotopic PDX models have become the roadblocks for the popularization of this tool;
3. A replacement of human stromal cells by mouse stroma occurs in the initial stage of PDX establishment [20], which blocks the study of the interaction between EC cells and stromal cells due to the loss of human stromal cells in PDX;
4. A lack of a functional immune system also prevents the analysis of immunotherapeutic approaches to EC therapy.

Although the drawbacks exist in the current EC-PDX models, the development of novel immunodeficient animals may help accelerate their usage in a preclinical study. For instance, tumor cells in immunodeficient Syrian hamster can communicate with host fibroblasts, which may provide growth factors to keep human cancer and stromal cells survive longer [163]. Moreover, humanized animal models with reconstituted human immune cells will be more meaningful, which allow the investigation of the interaction between cancer cells and various human immune cells.

## Search strategy

Searching databases include PubMed, Medline, and Web of Science by using “patient derived xenograft” and “esophagus*”, or “mouse avatar”, “xenograft”, “primary esophageal cancer”.

## References

[1] Bray F, Ferlay J, Soerjomataram I, Siegel RL, Torre LA, Jemal A. Global cancer statistics 2018: GLOBOCAN estimates of incidence and mortality worldwide for 36 cancers in 185 countries. CA Cancer J Clin. 2018; 68:394–424. 10.3322/caac.21492 30207593

[2] Malhotra GK, Yanala U, Ravipati A, Follet M, Vijayakumar M, Are C. Global trends in esophageal cancer. J Surg Oncol. 2017; 115:564–79. 10.1002/jso.24592 28320055

[3] Liu K, Zhao T, Wang J, Chen Y, Zhang R, Lan X, Que J. Etiology, cancer stem cells and potential diagnostic biomarkers for esophageal cancer. Cancer Lett. 2019; 458:21–28. 10.1016/j.canlet.2019.05.018 31125642 PMC6597177

[4] Abnet CC, Arnold M, Wei WQ. Epidemiology of Esophageal Squamous Cell Carcinoma. Gastroenterology. 2018; 154:360–73. 10.1053/j.gastro.2017.08.023 28823862 PMC5836473

[5] Taccioli C, Chen H, Jiang Y, Liu XP, Huang K, Smalley KJ, Farber JL, Croce CM, Fong LY. Dietary zinc deficiency fuels esophageal cancer development by inducing a distinct inflammatory signature. Oncogene. 2012; 31:4550–58. 10.1038/onc.2011.592 22179833 PMC3310953

[6] Chen Y, Tong Y, Yang C, Gan Y, Sun H, Bi H, Cao S, Yin X, Lu Z. Consumption of hot beverages and foods and the risk of esophageal cancer: a meta-analysis of observational studies. BMC Cancer. 2015; 15:449. 10.1186/s12885-015-1185-1 26031666 PMC4457273

[7] Wang RH. From reflux esophagitis to Barrett’s esophagus and esophageal adenocarcinoma. World J Gastroenterol. 2015; 21:5210–19. 10.3748/wjg.v21.i17.5210 25954094 PMC4419061

[8] Lin EW, Karakasheva TA, Hicks PD, Bass AJ, Rustgi AK. The tumor microenvironment in esophageal cancer. Oncogene. 2016; 35:5337–49. 10.1038/onc.2016.34 26923327 PMC5003768

[9] Arnold M, Soerjomataram I, Ferlay J, Forman D. Global incidence of oesophageal cancer by histological subtype in 2012. Gut. 2015; 64:381–87. 10.1136/gutjnl-2014-308124 25320104

[10] Gupta B, Kumar N. Worldwide incidence, mortality and time trends for cancer of the oesophagus. Eur J Cancer Prev. 2017; 26:107–18. 10.1097/CEJ.0000000000000249 27014938

[11] Domper Arnal MJ, Ferrández Arenas Á, Lanas Arbeloa Á. Esophageal cancer: Risk factors, screening and endoscopic treatment in Western and Eastern countries. World J Gastroenterol. 2015; 21:7933–43. 10.3748/wjg.v21.i26.7933 26185366 PMC4499337

[12] Huang FL, Yu SJ. Esophageal cancer: Risk factors, genetic association, and treatment. Asian J Surg. 2018; 41:210–15. 10.1016/j.asjsur.2016.10.005 27986415

[13] Madhavan A, Kamarajah SK, Navidi M, Wahed S, Immanuel A, Hayes N, Griffin SM, Phillips AW. The impact of age on patients undergoing transthoracic esophagectomy for cancer. Dis Esophagus. 2021; 34:doaa056. 10.1093/dote/doaa056 32556151

[14] Zeng Y, Liang W, Liu J, He J, Ng CS, Liu CC, Petersen RH, Rocco G, D’Amico T, Brunelli A, Chen H, Zhi X, Dong X, et al, and written on behalf of the AME Thoracic Surgery Collaborative Group. Esophageal cancer in elderly patients: a population-based study. J Thorac Dis. 2018; 10:448–57. 10.21037/jtd.2018.01.89 29600077 PMC5863173

[15] Veeranki OL, Tong Z, Mejia A, Verma A, Katkhuda R, Bassett R, Kim TB, Wang J, Lang W, Mino B, Solis L, Kingsley C, Norton W, et al. A novel patient-derived orthotopic xenograft model of esophageal adenocarcinoma provides a platform for translational discoveries. Dis Model Mech. 2019; 12:dmm041004. 10.1242/dmm.041004 31732509 PMC6918774

[16] Hori T, Yamashita Y, Ohira M, Matsumura Y, Muguruma K, Hirakawa K. A novel orthotopic implantation model of human esophageal carcinoma in nude rats: CD44H mediates cancer cell invasion *in vitro* and *in vivo*. Int J Cancer. 2001; 92:489–96. 10.1002/ijc.1234 11304682

[17] Ohara T, Takaoka M, Sakurama K, Nagaishi K, Takeda H, Shirakawa Y, Yamatsuji T, Nagasaka T, Matsuoka J, Tanaka N, Naomoto Y. The establishment of a new mouse model with orthotopic esophageal cancer showing the esophageal stricture. Cancer Lett. 2010; 293:207–12. 10.1016/j.canlet.2010.01.017 20153104

[18] Liu A, Zhu J, Wu G, Cao L, Tan Z, Zhang S, Jiang L, Wu J, Li M, Song L, Li J. Antagonizing miR-455-3p inhibits chemoresistance and aggressiveness in esophageal squamous cell carcinoma. Mol Cancer. 2017; 16:106. 10.1186/s12943-017-0669-9 28633632 PMC5479030

[19] Zhang J, Jiang D, Li X, Lv J, Xie L, Zheng L, Gavine PR, Hu Q, Shi Y, Tan L, Ge D, Xu S, Li L, et al. Establishment and characterization of esophageal squamous cell carcinoma patient-derived xenograft mouse models for preclinical drug discovery. Lab Invest. 2014; 94:917–26. 10.1038/labinvest.2014.77 24999713

[20] Damhofer H, Ebbing EA, Steins A, Welling L, Tol JA, Krishnadath KK, van Leusden T, van de Vijver MJ, Besselink MG, Busch OR, van Berge Henegouwen MI, van Delden O, Meijer SL, et al. Establishment of patient-derived xenograft models and cell lines for malignancies of the upper gastrointestinal tract. J Transl Med. 2015; 13:115. 10.1186/s12967-015-0469-1 25884700 PMC4419410

[21] Jiang Y, Wu Q, Yang X, Zhao J, Jin Y, Li K, Ma Y, Chen X, Tian F, Zhao S, Xu J, Lu J, Yin X, et al. A method for establishing a patient-derived xenograft model to explore new therapeutic strategies for esophageal squamous cell carcinoma. Oncol Rep. 2016; 35:785–92. 10.3892/or.2015.4459 26718633

[22] Zou J, Liu Y, Wang J, Liu Z, Lu Z, Chen Z, Li Z, Dong B, Huang W, Li Y, Gao J, Shen L. Establishment and genomic characterizations of patient-derived esophageal squamous cell carcinoma xenograft models using biopsies for treatment optimization. J Transl Med. 2018; 16:15. 10.1186/s12967-018-1379-9 29370817 PMC5785825

[23] Szadvari I, Krizanova O, Babula P. Athymic nude mice as an experimental model for cancer treatment. Physiol Res. 2016 (Suppl 4); 65:S441–53. 10.33549/physiolres.933526 28006926

[24] Budzynski W, Radzikowski C. Cytotoxic cells in immunodeficient athymic mice. Immunopharmacol Immunotoxicol. 1994; 16:319–46. 10.3109/08923979409007097 7528237

[25] Taghian A, Budach W, Zietman A, Freeman J, Gioioso D, Ruka W, Suit HD. Quantitative comparison between the transplantability of human and murine tumors into the subcutaneous tissue of NCr/Sed-nu/nu nude and severe combined immunodeficient mice. Cancer Res. 1993; 53:5012–17. 8402692

[26] Shultz LD, Schweitzer PA, Christianson SW, Gott B, Schweitzer IB, Tennent B, McKenna S, Mobraaten L, Rajan TV, Greiner DL. Multiple defects in innate and adaptive immunologic function in NOD/LtSz-scid mice. J Immunol. 1995; 154:180–91. 7995938

[27] Dodbiba L, Teichman J, Fleet A, Thai H, Sun B, Panchal D, Patel D, Tse A, Chen Z, Faluyi OO, Renouf DJ, Girgis H, Bandarchi B, et al. Primary esophageal and gastro-esophageal junction cancer xenograft models: clinicopathological features and engraftment. Lab Invest. 2013; 93:397–407. 10.1038/labinvest.2013.8 23399854

[28] Ito M, Kobayashi K, Nakahata T. NOD/Shi-scid IL2rgamma(null) (NOG) mice more appropriate for humanized mouse models. Curr Top Microbiol Immunol. 2008; 324:53–76. 10.1007/978-3-540-75647-7_3 18481452

[29] Ito M, Hiramatsu H, Kobayashi K, Suzue K, Kawahata M, Hioki K, Ueyama Y, Koyanagi Y, Sugamura K, Tsuji K, Heike T, Nakahata T. NOD/SCID/gamma(c)(null) mouse: an excellent recipient mouse model for engraftment of human cells. Blood. 2002; 100:3175–82. 10.1182/blood-2001-12-0207 12384415

[30] Huang P, Westmoreland SV, Jain RK, Fukumura D. Spontaneous nonthymic tumors in SCID mice. Comp Med. 2011; 61:227–34. 21819692 PMC3123755

[31] Shultz LD, Lyons BL, Burzenski LM, Gott B, Chen X, Chaleff S, Kotb M, Gillies SD, King M, Mangada J, Greiner DL, Handgretinger R. Human lymphoid and myeloid cell development in NOD/LtSz-scid IL2R gamma null mice engrafted with mobilized human hemopoietic stem cells. J Immunol. 2005; 174:6477–89. 10.4049/jimmunol.174.10.6477 15879151

[32] Santagostino SF, Arbona RJ, Nashat MA, White JR, Monette S. Pathology of Aging in NOD scid gamma Female Mice. Vet Pathol. 2017; 54:855–69. 10.1177/0300985817698210 28355107 PMC5548647

[33] Read M, Liu D, Duong CP, Cullinane C, Murray WK, Fennell CM, Shortt J, Westerman D, Burton P, Clemons NJ, Phillips WA. Intramuscular Transplantation Improves Engraftment Rates for Esophageal Patient-Derived Tumor Xenografts. Ann Surg Oncol. 2016; 23:305–11. 10.1245/s10434-015-4425-3 25691278

[34] Coomer AR, Farese JP, Milner R, Taylor D, Salute ME, Rajon DA, Bova FJ, Siemann DW. Development of an intramuscular xenograft model of canine osteosarcoma in mice for evaluation of the effects of radiation therapy. Am J Vet Res. 2009; 70:127–33. 10.2460/ajvr.70.1.127 19119958

[35] Oktay K. Ovarian tissue cryopreservation and transplantation: preliminary findings and implications for cancer patients. Hum Reprod Update. 2001; 7:526–34. 10.1093/humupd/7.6.526 11727860

[36] Zhu H, Wang C, Wang J, Chen D, Deng J, Deng J, Fan J, Badakhshi H, Huang X, Zhang L, Cai J, Guo S, Qian W, et al. A subset of esophageal squamous cell carcinoma patient-derived xenografts respond to cetuximab, which is predicted by high EGFR expression and amplification. J Thorac Dis. 2018; 10:5328–38. 10.21037/jtd.2018.09.18 30416780 PMC6196161

[37] Su D, Zhang D, Jin J, Ying L, Han M, Chen K, Li B, Wu J, Xie Z, Zhang F, Lin Y, Cheng G, Li JY, et al. Identification of predictors of drug sensitivity using patient-derived models of esophageal squamous cell carcinoma. Nat Commun. 2019; 10:5076. 10.1038/s41467-019-12846-7 31700061 PMC6838071

[38] Cameron D, Piccart-Gebhart MJ, Gelber RD, Procter M, Goldhirsch A, de Azambuja E, Castro G Jr, Untch M, Smith I, Gianni L, Baselga J, Al-Sakaff N, Lauer S, et al, and Herceptin Adjuvant (HERA) Trial Study Team. 11 years’ follow-up of trastuzumab after adjuvant chemotherapy in HER2-positive early breast cancer: final analysis of the HERceptin Adjuvant (HERA) trial. Lancet. 2017; 389:1195–205. 10.1016/S0140-6736(16)32616-2 28215665 PMC5465633

[39] Wu X, Zhang J, Zhen R, Lv J, Zheng L, Su X, Zhu G, Gavine PR, Xu S, Lu S, Hou J, Liu Y, Xu C, et al. Trastuzumab anti-tumor efficacy in patient-derived esophageal squamous cell carcinoma xenograft (PDECX) mouse models. J Transl Med. 2012; 10:180. 10.1186/1479-5876-10-180 22935382 PMC3485623

[40] Karakasheva TA, Lin EW, Tang Q, Qiao E, Waldron TJ, Soni M, Klein-Szanto AJ, Sahu V, Basu D, Ohashi S, Baba K, Giaccone ZT, Walker SR, et al. IL-6 Mediates Cross-Talk between Tumor Cells and Activated Fibroblasts in the Tumor Microenvironment. Cancer Res. 2018; 78:4957–70. 10.1158/0008-5472.CAN-17-2268 29976575 PMC6125177

[41] Ebbing EA, van der Zalm AP, Steins A, Creemers A, Hermsen S, Rentenaar R, Klein M, Waasdorp C, Hooijer GK, Meijer SL, Krishnadath KK, Punt CJ, van Berge Henegouwen MI, et al. Stromal-derived interleukin 6 drives epithelial-to-mesenchymal transition and therapy resistance in esophageal adenocarcinoma. Proc Natl Acad Sci USA. 2019; 116:2237–42. 10.1073/pnas.1820459116 30670657 PMC6369811

[42] Ren Y, Zheng J, Fan S, Wang L, Cheng M, Shi D, Zhang W, Tang R, Yu Y, Jiao L, Ni J, Yang H, Cai H, et al. Anti-tumor efficacy of theliatinib in esophageal cancer patient-derived xenografts models with epidermal growth factor receptor (EGFR) overexpression and gene amplification. Oncotarget. 2017; 8:50832–44. 10.18632/oncotarget.17243 28881608 PMC5584209

[43] Dodbiba L, Teichman J, Fleet A, Thai H, Starmans MH, Navab R, Chen Z, Girgis H, Eng L, Espin-Garcia O, Shen X, Bandarchi B, Schwock J, et al. Appropriateness of using patient-derived xenograft models for pharmacologic evaluation of novel therapies for esophageal/gastro-esophageal junction cancers. PLoS One. 2015; 10:e0121872. 10.1371/journal.pone.0121872 25826681 PMC4380353

[44] Stewart E, Federico SM, Chen X, Shelat AA, Bradley C, Gordon B, Karlstrom A, Twarog NR, Clay MR, Bahrami A, Freeman BB 3rd, Xu B, Zhou X, et al. Orthotopic patient-derived xenografts of paediatric solid tumours. Nature. 2017; 549:96–100. 10.1038/nature23647 28854174 PMC5659286

[45] Prasetyanti PR, van Hooff SR, van Herwaarden T, de Vries N, Kalloe K, Rodermond H, van Leersum R, de Jong JH, Franitza M, Nürnberg P, Todaro M, Stassi G, Medema JP. Capturing colorectal cancer inter-tumor heterogeneity in patient-derived xenograft (PDX) models. Int J Cancer. 2019; 144:366–71. 10.1002/ijc.31767 30151914 PMC6587871

[46] Lange T, Oh-Hohenhorst SJ, Joosse SA, Pantel K, Hahn O, Gosau T, Dyshlovoy SA, Wellbrock J, Feldhaus S, Maar H, Gehrcke R, Kluth M, Simon R, et al. Development and Characterization of a Spontaneously Metastatic Patient-Derived Xenograft Model of Human Prostate Cancer. Sci Rep. 2018; 8:17535. 10.1038/s41598-018-35695-8 30510249 PMC6277427

[47] Gebski V, Burmeister B, Smithers BM, Foo K, Zalcberg J, Simes J, and Australasian Gastro-Intestinal Trials Group. Survival benefits from neoadjuvant chemoradiotherapy or chemotherapy in oesophageal carcinoma: a meta-analysis. Lancet Oncol. 2007; 8:226–34. 10.1016/S1470-2045(07)70039-6 17329193

[48] Blencowe NS, McNair AG, Davis CR, Brookes ST, Blazeby JM. Standards of outcome reporting in surgical oncology: a case study in esophageal cancer. Ann Surg Oncol. 2012; 19:4012–18. 10.1245/s10434-012-2497-x 22820935

[49] Geng J, Yuan X, Wei M, Wu J, Qin ZH. The diverse role of TIGAR in cellular homeostasis and cancer. Free Radic Res. 2018; 52:1240–49. 10.1080/10715762.2018.1489133 30284488

[50] GongSun X, Zhao Y, Jiang B, Xin Z, Shi M, Song L, Qin Q, Wang Q, Liu X. Inhibition of MUC1-C regulates metabolism by AKT pathway in esophageal squamous cell carcinoma. J Cell Physiol. 2019; 234:12019–28. 10.1002/jcp.27863 30523643 PMC6587484

[51] Chu J, Niu X, Chang J, Shao M, Peng L, Xi Y, Lin A, Wang C, Cui Q, Luo Y, Fan W, Chen Y, Sun Y, et al. Metabolic remodeling by TIGAR overexpression is a therapeutic target in esophageal squamous-cell carcinoma. Theranostics. 2020; 10:3488–502. 10.7150/thno.41427 32206103 PMC7069087

[52] Ross D, Siegel D. Functions of NQO1 in Cellular Protection and CoQ_10_ Metabolism and its Potential Role as a Redox Sensitive Molecular Switch. Front Physiol. 2017; 8:595. 10.3389/fphys.2017.00595 28883796 PMC5573868

[53] Mizumoto A, Ohashi S, Kamada M, Saito T, Nakai Y, Baba K, Hirohashi K, Mitani Y, Kikuchi O, Matsubara J, Yamada A, Takahashi T, Lee H, et al. Combination treatment with highly bioavailable curcumin and NQO1 inhibitor exhibits potent antitumor effects on esophageal squamous cell carcinoma. J Gastroenterol. 2019; 54:687–98. 10.1007/s00535-019-01549-x 30737573 PMC6647399

[54] Gilmour DS, Pflugfelder G, Wang JC, Lis JT. Topoisomerase I interacts with transcribed regions in Drosophila cells. Cell. 1986; 44:401–07. 10.1016/0092-8674(86)90461-7 3002635

[55] Li M, Liu Y. Topoisomerase I in Human Disease Pathogenesis and Treatments. Genomics Proteomics Bioinformatics. 2016; 14:166–71. 10.1016/j.gpb.2016.02.004 27181710 PMC4936607

[56] Pourquier P, Pommier Y. Topoisomerase I-mediated DNA damage. Adv Cancer Res. 2001; 80:189–216. 10.1016/S0065-230X(01)80016-6 11034544

[57] Leppard JB, Champoux JJ. Human DNA topoisomerase I: relaxation, roles, and damage control. Chromosoma. 2005; 114:75–85. 10.1007/s00412-005-0345-5 15830206

[58] Hanagiri T, Ono K, Kuwata T, Takenaka M, Oka S, Chikaishi Y, Shigematsu Y, Nagata Y, Shimokawa H, Nakagawa M, Uramoto H, So T, Tanaka F. Evaluation of topoisomerase I/topoisomerase IIalpha status in esophageal cancer. J UOEH. 2011; 33:205–16. 10.7888/juoeh.33.205 21913377

[59] Song M, Yin S, Zhao R, Liu K, Kundu JK, Shim JH, Lee MH, Dong Z. (S)-10-Hydroxycamptothecin Inhibits Esophageal Squamous Cell Carcinoma Growth *In Vitro* and *In Vivo* Via Decreasing Topoisomerase I Enzyme Activity. Cancers (Basel). 2019; 11:1964. 10.3390/cancers11121964 31817790 PMC6966462

[60] Pratesi G, Beretta GL, Zunino F. Gimatecan, a novel camptothecin with a promising preclinical profile. Anticancer Drugs. 2004; 15:545–52. 10.1097/01.cad.0000131687.08175.14 15205595

[61] Zou J, Li S, Chen Z, Lu Z, Gao J, Zou J, Lin X, Li Y, Zhang C, Shen L. A novel oral camptothecin analog, gimatecan, exhibits superior antitumor efficacy than irinotecan toward esophageal squamous cell carcinoma *in vitro* and *in vivo*. Cell Death Dis. 2018; 9:661. 10.1038/s41419-018-0700-0 29855512 PMC5981453

[62] Wang Z. ErbB Receptors and Cancer. Methods Mol Biol. 2017; 1652:3–35. 10.1007/978-1-4939-7219-7_1 28791631

[63] Roskoski R Jr. Small molecule inhibitors targeting the EGFR/ErbB family of protein-tyrosine kinases in human cancers. Pharmacol Res. 2019; 139:395–411. 10.1016/j.phrs.2018.11.014 30500458

[64] Arteaga CL, Engelman JA. ERBB receptors: from oncogene discovery to basic science to mechanism-based cancer therapeutics. Cancer Cell. 2014; 25:282–303. 10.1016/j.ccr.2014.02.025 24651011 PMC4018830

[65] Zhang W, Zhu H, Liu X, Wang Q, Zhang X, He J, Sun K, Liu X, Zhou Z, Xu N, Xiao Z. Epidermal growth factor receptor is a prognosis predictor in patients with esophageal squamous cell carcinoma. Ann Thorac Surg. 2014; 98:513–19. 10.1016/j.athoracsur.2014.03.015 24881860

[66] Wang WJ, Wu MJ, Chang J, Xie P, Lu ZQ, Ren JL. Role of human epidermal growth factor receptor 2 as a prognostic factor for survival in esophageal carcinoma: evidence from 2872 subjects. Minerva Med. 2016; 107:328–41. 27309036

[67] Goldstein NI, Prewett M, Zuklys K, Rockwell P, Mendelsohn J. Biological efficacy of a chimeric antibody to the epidermal growth factor receptor in a human tumor xenograft model. Clin Cancer Res. 1995; 1:1311–18. 9815926

[68] Bang YJ, Van Cutsem E, Feyereislova A, Chung HC, Shen L, Sawaki A, Lordick F, Ohtsu A, Omuro Y, Satoh T, Aprile G, Kulikov E, Hill J, et al, and ToGA Trial Investigators. Trastuzumab in combination with chemotherapy versus chemotherapy alone for treatment of HER2-positive advanced gastric or gastro-oesophageal junction cancer (ToGA): a phase 3, open-label, randomised controlled trial. Lancet. 2010; 376:687–97. 10.1016/S0140-6736(10)61121-X 20728210

[69] Doi T, Shitara K, Naito Y, Shimomura A, Fujiwara Y, Yonemori K, Shimizu C, Shimoi T, Kuboki Y, Matsubara N, Kitano A, Jikoh T, Lee C, et al. Safety, pharmacokinetics, and antitumour activity of trastuzumab deruxtecan (DS-8201), a HER2-targeting antibody-drug conjugate, in patients with advanced breast and gastric or gastro-oesophageal tumours: a phase 1 dose-escalation study. Lancet Oncol. 2017; 18:1512–22. 10.1016/S1470-2045(17)30604-6 29037983

[70] Ebbing EA, Medema JP, Damhofer H, Meijer SL, Krishnadath KK, van Berge Henegouwen MI, Bijlsma MF, van Laarhoven HW. ADAM10-mediated release of heregulin confers resistance to trastuzumab by activating HER3. Oncotarget. 2016; 7:10243–54. 10.18632/oncotarget.7200 26863569 PMC4891117

[71] Oh IJ, Hur JY, Park CK, Kim YC, Kim SJ, Lee MK, Kim HJ, Lee KY, Lee JC, Choi CM. Clinical Activity of Pan-HER Inhibitors Against HER2-Mutant Lung Adenocarcinoma. Clin Lung Cancer. 2018; 19:e775–81. 10.1016/j.cllc.2018.05.018 30149884

[72] Duchnowska R, Loibl S, Jassem J. Tyrosine kinase inhibitors for brain metastases in HER2-positive breast cancer. Cancer Treat Rev. 2018; 67:71–77. 10.1016/j.ctrv.2018.05.004 29772459

[73] Liu Z, Chen Z, Wang J, Zhang M, Li Z, Wang S, Dong B, Zhang C, Gao J, Shen L. Mouse avatar models of esophageal squamous cell carcinoma proved the potential for EGFR-TKI afatinib and uncovered Src family kinases involved in acquired resistance. J Hematol Oncol. 2018; 11:109. 10.1186/s13045-018-0651-z 30157900 PMC6114252

[74] Sanchez-Vega F, Hechtman JF, Castel P, Ku GY, Tuvy Y, Won H, Fong CJ, Bouvier N, Nanjangud GJ, Soong J, Vakiani E, Schattner M, Kelsen DP, et al. EGFR and MET Amplifications Determine Response to HER2 Inhibition in ERBB2-Amplified Esophagogastric Cancer. Cancer Discov. 2019; 9:199–209. 10.1158/2159-8290.CD-18-0598 30463996 PMC6368868

[75] Hou W, Qin X, Zhu X, Fei M, Liu P, Liu L, Moon H, Zhang P, Greshock J, Bachman KE, Ye BC, Wang H, Zang CY. Lapatinib inhibits the growth of esophageal squamous cell carcinoma and synergistically interacts with 5-fluorouracil in patient-derived xenograft models. Oncol Rep. 2013; 30:707–14. 10.3892/or.2013.2500 23708506

[76] Huang Y, Li T, Ems-McClung SC, Walczak CE, Prigent C, Zhu X, Zhang X, Zheng Y. Aurora A activation in mitosis promoted by BuGZ. J Cell Biol. 2018; 217:107–16. 10.1083/jcb.201706103 29074706 PMC5748987

[77] Goldenson B, Crispino JD. The aurora kinases in cell cycle and leukemia. Oncogene. 2015; 34:537–45. 10.1038/onc.2014.14 24632603 PMC4167158

[78] Tong T, Zhong Y, Kong J, Dong L, Song Y, Fu M, Liu Z, Wang M, Guo L, Lu S, Wu M, Zhan Q. Overexpression of Aurora-A contributes to malignant development of human esophageal squamous cell carcinoma. Clin Cancer Res. 2004; 10:7304–10. 10.1158/1078-0432.CCR-04-0806 15534106

[79] Lin YS, Su LJ, Yu CT, Wong FH, Yeh HH, Chen SL, Wu JC, Lin WJ, Shiue YL, Liu HS, Hsu SL, Lai JM, Huang CY. Gene expression profiles of the aurora family kinases. Gene Expr. 2006; 13:15–26. 10.3727/000000006783991962 16572587 PMC6032448

[80] Wang X, Lu N, Niu B, Chen X, Xie J, Cheng N. Overexpression of Aurora-A enhances invasion and matrix metalloproteinase-2 expression in esophageal squamous cell carcinoma cells. Mol Cancer Res. 2012; 10:588–96. 10.1158/1541-7786.MCR-11-0416 22522455

[81] Yu X, Liang Q, Liu W, Zhou L, Li W, Liu H. Deguelin, an Aurora B Kinase Inhibitor, Exhibits Potent Anti-Tumor Effect in Human Esophageal Squamous Cell Carcinoma. EBioMedicine. 2017; 26:100–11. 10.1016/j.ebiom.2017.10.030 29129699 PMC5832566

[82] Jin G, Yao K, Guo Z, Zhao Z, Liu K, Liu F, Chen H, Gorja DR, Reddy K, Bode AM, Dong Z, Dong Z. APIO-EE-9 is a novel Aurora A and B antagonist that suppresses esophageal cancer growth in a PDX mouse model. Oncotarget. 2017; 8:53387–404. 10.18632/oncotarget.18508 28881819 PMC5581118

[83] Klein ME, Kovatcheva M, Davis LE, Tap WD, Koff A. CDK4/6 Inhibitors: The Mechanism of Action May Not Be as Simple as Once Thought. Cancer Cell. 2018; 34:9–20. 10.1016/j.ccell.2018.03.023 29731395 PMC6039233

[84] Hamilton E, Infante JR. Targeting CDK4/6 in patients with cancer. Cancer Treat Rev. 2016; 45:129–38. 10.1016/j.ctrv.2016.03.002 27017286

[85] Pernas S, Tolaney SM, Winer EP, Goel S. CDK4/6 inhibition in breast cancer: current practice and future directions. Ther Adv Med Oncol. 2018; 10:1758835918786451. 10.1177/1758835918786451 30038670 PMC6050811

[86] Li F, Xu Y, Liu B, Singh PK, Zhao W, Jin J, Han G, Scott AW, Dong X, Huo L, Ma L, Pizzi MP, Wang Y, et al. YAP1-Mediated CDK6 Activation Confers Radiation Resistance in Esophageal Cancer - Rationale for the Combination of YAP1 and CDK4/6 Inhibitors in Esophageal Cancer. Clin Cancer Res. 2019; 25:2264–77. 10.1158/1078-0432.CCR-18-1029 30563933

[87] Xu Y, Li X, Xu YF, Yang XM. Poorly Differentiated Esophageal Neuroendocrine Carcinoma Treated With the CDK4/6 Inhibitor, Palbociclib: A Case Report and Literature Review. Am J Ther. 2018; 25:e595–98. 10.1097/MJT.0000000000000610 29232286

[88] Doi T, Hewes B, Kakizume T, Tajima T, Ishikawa N, Yamada Y. Phase I study of single-agent ribociclib in Japanese patients with advanced solid tumors. Cancer Sci. 2018; 109:193–98. 10.1111/cas.13428 29059492 PMC5765307

[89] Frankell AM, Jammula S, Li X, Contino G, Killcoyne S, Abbas S, Perner J, Bower L, Devonshire G, Ococks E, Grehan N, Mok J, O’Donovan M, et al, and Oesophageal Cancer Clinical and Molecular Stratification (OCCAMS) Consortium. The landscape of selection in 551 esophageal adenocarcinomas defines genomic biomarkers for the clinic. Nat Genet. 2019; 51:506–16. 10.1038/s41588-018-0331-5 30718927 PMC6420087

[90] Wang J, Li Q, Yuan J, Wang J, Chen Z, Liu Z, Li Z, Lai Y, Gao J, Shen L. CDK4/6 inhibitor-SHR6390 exerts potent antitumor activity in esophageal squamous cell carcinoma by inhibiting phosphorylated Rb and inducing G1 cell cycle arrest. J Transl Med. 2017; 15:127. 10.1186/s12967-017-1231-7 28578693 PMC5457542

[91] Tong Z, Chatterjee D, Deng D, Veeranki O, Mejia A, Ajani JA, Hofstetter W, Lin S, Guha S, Kopetz S, Krishnan S, Maru D. Antitumor effects of cyclin dependent kinase 9 inhibition in esophageal adenocarcinoma. Oncotarget. 2017; 8:28696–710. 10.18632/oncotarget.15645 28404924 PMC5438684

[92] Veeranki OL, Tong Z, Dokey R, Mejia A, Zhang J, Qiao Y, Singh PK, Katkhuda R, Mino B, Tailor R, Canales JR, Bassett R, Ajani J, et al. Targeting cyclin-dependent kinase 9 by a novel inhibitor enhances radiosensitization and identifies Axl as a novel downstream target in esophageal adenocarcinoma. Oncotarget. 2019; 10:4703–18. 10.18632/oncotarget.27095 31384397 PMC6659793

[93] You Z, Xu D, Ji J, Guo W, Zhu W, He J. JAK/STAT signal pathway activation promotes progression and survival of human oesophageal squamous cell carcinoma. Clin Transl Oncol. 2012; 14:143–49. 10.1007/s12094-012-0774-6 22301404

[94] Andl CD, Mizushima T, Oyama K, Bowser M, Nakagawa H, Rustgi AK. EGFR-induced cell migration is mediated predominantly by the JAK-STAT pathway in primary esophageal keratinocytes. Am J Physiol Gastrointest Liver Physiol. 2004; 287:G1227–37. 10.1152/ajpgi.00253.2004 15284024

[95] Groner B, von Manstein V. Jak Stat signaling and cancer: Opportunities, benefits and side effects of targeted inhibition. Mol Cell Endocrinol. 2017; 451:1–14. 10.1016/j.mce.2017.05.033 28576744

[96] Ying J, Qiu X, Lu Y, Zhang M. SOCS1 and its Potential Clinical Role in Tumor. Pathol Oncol Res. 2019; 25:1295–301. 10.1007/s12253-019-00612-5 30761449

[97] Yasukawa H, Misawa H, Sakamoto H, Masuhara M, Sasaki A, Wakioka T, Ohtsuka S, Imaizumi T, Matsuda T, Ihle JN, Yoshimura A. The JAK-binding protein JAB inhibits Janus tyrosine kinase activity through binding in the activation loop. EMBO J. 1999; 18:1309–20. 10.1093/emboj/18.5.1309 10064597 PMC1171221

[98] Sugase T, Takahashi T, Serada S, Nakatsuka R, Fujimoto M, Ohkawara T, Hara H, Nishigaki T, Tanaka K, Miyazaki Y, Makino T, Kurokawa Y, Yamasaki M, et al. Suppressor of cytokine signaling-1 gene therapy induces potent antitumor effect in patient-derived esophageal squamous cell carcinoma xenograft mice. Int J Cancer. 2017; 140:2608–21. 10.1002/ijc.30666 28233302

[99] Yang Y, Jin G, Liu H, Liu K, Zhao J, Chen X, Wang D, Bai R, Li X, Jang Y, Lu J, Xing Y, Dong Z. Metformin inhibits esophageal squamous cell carcinoma-induced angiogenesis by suppressing JAK/STAT3 signaling pathway. Oncotarget. 2017; 8:74673–87. 10.18632/oncotarget.20341 29088816 PMC5650371

[100] Tian F, Yang X, Liu Y, Yuan X, Fan T, Zhang F, Zhao J, Lu J, Jiang Y, Dong Z, Yang Y. Constitutive activated STAT3 is an essential regulator and therapeutic target in esophageal squamous cell carcinoma. Oncotarget. 2017; 8:88719–29. 10.18632/oncotarget.20838 29179470 PMC5687640

[101] De Luca A, Maiello MR, D’Alessio A, Pergameno M, Normanno N. The RAS/RAF/MEK/ERK and the PI3K/AKT signalling pathways: role in cancer pathogenesis and implications for therapeutic approaches. Expert Opin Ther Targets. 2012 (Suppl 2); 16:S17–27. 10.1517/14728222.2011.639361 22443084

[102] Nan P, Wang T, Li C, Li H, Wang J, Zhang J, Dou N, Zhan Q, Ma F, Wang H, Qian H. MTA1 promotes tumorigenesis and development of esophageal squamous cell carcinoma via activating the MEK/ERK/p90RSK signaling pathway. Carcinogenesis. 2020; 41:1263–72. 10.1093/carcin/bgz200 31783401

[103] Riquelme E, Behrens C, Lin HY, Simon G, Papadimitrakopoulou V, Izzo J, Moran C, Kalhor N, Lee JJ, Minna JD, Wistuba II. Modulation of EZH2 Expression by MEK-ERK or PI3K-AKT Signaling in Lung Cancer Is Dictated by Different KRAS Oncogene Mutations. Cancer Res. 2016; 76:675–85. 10.1158/0008-5472.CAN-15-1141 26676756 PMC4738155

[104] Ebi H, Costa C, Faber AC, Nishtala M, Kotani H, Juric D, Della Pelle P, Song Y, Yano S, Mino-Kenudson M, Benes CH, Engelman JA. PI3K regulates MEK/ERK signaling in breast cancer via the Rac-GEF, P-Rex1. Proc Natl Acad Sci USA. 2013; 110:21124–29. 10.1073/pnas.1314124110 24327733 PMC3876254

[105] Samatar AA, Poulikakos PI. Targeting RAS-ERK signalling in cancer: promises and challenges. Nat Rev Drug Discov. 2014; 13:928–42. 10.1038/nrd4281 25435214

[106] Roberts PJ, Der CJ. Targeting the Raf-MEK-ERK mitogen-activated protein kinase cascade for the treatment of cancer. Oncogene. 2007; 26:3291–310. 10.1038/sj.onc.1210422 17496923

[107] Xie X, Zu X, Liu F, Wang T, Wang X, Chen H, Liu K, Wang P, Liu F, Zheng Y, Bode AM, Dong Z, Kim DJ. Purpurogallin is a novel mitogen-activated protein kinase kinase 1/2 inhibitor that suppresses esophageal squamous cell carcinoma growth *in vitro* and *in vivo*. Mol Carcinog. 2019; 58:1248–59. 10.1002/mc.23007 31100197

[108] Kim WH, Song HO, Choi HJ, Bang HI, Choi DY, Park H. Ethyl gallate induces apoptosis of HL-60 cells by promoting the expression of caspases-8, -9, -3, apoptosis-inducing factor and endonuclease G. Int J Mol Sci. 2012; 13:11912–22. 10.3390/ijms130911912 23109891 PMC3472783

[109] Wang HR, Sui HC, Ding YY, Zhu BT. Stimulation of the Production of Prostaglandin E_2_ by Ethyl Gallate, a Natural Phenolic Compound Richly Contained in Longan. Biomolecules. 2018; 8:91. 10.3390/biom8030091 30200641 PMC6165217

[110] Kalaivani T, Rajasekaran C, Mathew L. Free radical scavenging, cytotoxic, and hemolytic activities of an active antioxidant compound ethyl gallate from leaves of Acacia nilotica (L.) Wild. Ex. Delile subsp. indica (Benth.) Brenan. J Food Sci. 2011; 76:T144–49. 10.1111/j.1750-3841.2011.02243.x 22417526

[111] Liu F, Zu X, Xie X, Liu K, Chen H, Wang T, Liu F, Bode AM, Zheng Y, Dong Z, Kim DJ. Ethyl gallate as a novel ERK1/2 inhibitor suppresses patient-derived esophageal tumor growth. Mol Carcinog. 2019; 58:533–43. 10.1002/mc.22948 30499613

[112] Wiggin GR, Soloaga A, Foster JM, Murray-Tait V, Cohen P, Arthur JS. MSK1 and MSK2 are required for the mitogen- and stress-induced phosphorylation of CREB and ATF1 in fibroblasts. Mol Cell Biol. 2002; 22:2871–81. 10.1128/MCB.22.8.2871-2881.2002 11909979 PMC133730

[113] Soloaga A, Thomson S, Wiggin GR, Rampersaud N, Dyson MH, Hazzalin CA, Mahadevan LC, Arthur JS. MSK2 and MSK1 mediate the mitogen- and stress-induced phosphorylation of histone H3 and HMG-14. EMBO J. 2003; 22:2788–97. 10.1093/emboj/cdg273 12773393 PMC156769

[114] Zhang C, Zhang J, Wu Q, Xu B, Jin G, Qiao Y, Zhao S, Yang Y, Shang J, Li X, Liu K. Sulforaphene induces apoptosis and inhibits the invasion of esophageal cancer cells through MSK2/CREB/Bcl-2 and cadherin pathway *in vivo* and *in vitro*. Cancer Cell Int. 2019; 19:342. 10.1186/s12935-019-1061-1 31889894 PMC6921404

[115] Xie X, Liu K, Liu F, Chen H, Wang X, Zu X, Ma X, Wang T, Wu Q, Zheng Y, Bode AM, Dong Z, Kim DJ. Gossypetin is a novel MKK3 and MKK6 inhibitor that suppresses esophageal cancer growth *in vitro* and *in vivo*. Cancer Lett. 2019; 442:126–36. 10.1016/j.canlet.2018.10.016 30391783

[116] Lund ME, Campbell DH, Walsh BJ. The Role of Glypican-1 in the Tumour Microenvironment. Adv Exp Med Biol. 2020; 1245:163–76. 10.1007/978-3-030-40146-7_8 32266658

[117] Hara H, Takahashi T, Serada S, Fujimoto M, Ohkawara T, Nakatsuka R, Harada E, Nishigaki T, Takahashi Y, Nojima S, Miyazaki Y, Makino T, Kurokawa Y, et al. Overexpression of glypican-1 implicates poor prognosis and their chemoresistance in oesophageal squamous cell carcinoma. Br J Cancer. 2016; 115:66–75. 10.1038/bjc.2016.183 27310703 PMC4931380

[118] Li J, Chen Y, Zhan C, Zhu J, Weng S, Dong L, Liu T, Shen X. Glypican-1 Promotes Tumorigenesis by Regulating the PTEN/Akt/β-Catenin Signaling Pathway in Esophageal Squamous Cell Carcinoma. Dig Dis Sci. 2019; 64:1493–502. 10.1007/s10620-019-5461-9 30730015

[119] Harada E, Serada S, Fujimoto M, Takahashi Y, Takahashi T, Hara H, Nakatsuka R, Sugase T, Nishigaki T, Saito Y, Hiramatsu K, Nojima S, Mitsuo R, et al. Glypican-1 targeted antibody-based therapy induces preclinical antitumor activity against esophageal squamous cell carcinoma. Oncotarget. 2017; 8:24741–52. 10.18632/oncotarget.15799 28445969 PMC5421884

[120] De Luca A, Cerrato V, Fucà E, Parmigiani E, Buffo A, Leto K. Sonic hedgehog patterning during cerebellar development. Cell Mol Life Sci. 2016; 73:291–303. 10.1007/s00018-015-2065-1 26499980 PMC11108499

[121] Cortes JE, Gutzmer R, Kieran MW, Solomon JA. Hedgehog signaling inhibitors in solid and hematological cancers. Cancer Treat Rev. 2019; 76:41–50. 10.1016/j.ctrv.2019.04.005 31125907

[122] van den Brink GR. Hedgehog signaling in development and homeostasis of the gastrointestinal tract. Physiol Rev. 2007; 87:1343–75. 10.1152/physrev.00054.2006 17928586

[123] Alvarez-Trotta A, Wang Z, Shersher E, Li B, Long J, Lohse I, Wahlestedt C, El-Rifai W, Robbins DJ, Capobianco AJ. The bromodomain inhibitor IBET-151 attenuates vismodegib-resistant esophageal adenocarcinoma growth through reduction of GLI signaling. Oncotarget. 2020; 11:3174–87. 10.18632/oncotarget.27699 32913560 PMC7443367

[124] Teichman J, Dodbiba L, Thai H, Fleet A, Morey T, Liu L, McGregor M, Cheng D, Chen Z, Darling G, Brhane Y, Song Y, Espin-Garcia O, et al. Hedgehog inhibition mediates radiation sensitivity in mouse xenograft models of human esophageal adenocarcinoma. PLoS One. 2018; 13:e0194809. 10.1371/journal.pone.0194809 29715275 PMC5929523

[125] Yang L, Wang LS, Chen XL, Gatalica Z, Qiu S, Liu Z, Stoner G, Zhang H, Weiss H, Xie J. Hedgehog signaling activation in the development of squamous cell carcinoma and adenocarcinoma of esophagus. Int J Biochem Mol Biol. 2012; 3:46–57. 22509480 PMC3325770

[126] Kelly RJ, Ansari AM, Miyashita T, Zahurak M, Lay F, Ahmed AK, Born LJ, Pezhouh MK, Salimian KJ, Ng C, Matsangos AE, Stricker-Krongrad AH, Mukaisho KI, et al. Targeting the Hedgehog Pathway Using Itraconazole to Prevent Progression of Barrett’s Esophagus to Invasive Esophageal Adenocarcinoma. Ann Surg. 2019. [Epub ahead of print]. 10.1097/SLA.0000000000003455 31290765 PMC8147663

[127] Wang L, Jin JQ, Zhou Y, Tian Z, Jablons DM, He B. Gli is activated and promotes epithelial-mesenchymal transition in human esophageal adenocarcinoma. Oncotarget. 2017; 9:853–65. 10.18632/oncotarget.22856 29416661 PMC5787518

[128] Sims-Mourtada J, Izzo JG, Apisarnthanarax S, Wu TT, Malhotra U, Luthra R, Liao Z, Komaki R, van der Kogel A, Ajani J, Chao KS. Hedgehog: an attribute to tumor regrowth after chemoradiotherapy and a target to improve radiation response. Clin Cancer Res. 2006; 12:6565–72. 10.1158/1078-0432.CCR-06-0176 17085672

[129] Carnero A, Blanco-Aparicio C, Renner O, Link W, Leal JF. The PTEN/PI3K/AKT signalling pathway in cancer, therapeutic implications. Curr Cancer Drug Targets. 2008; 8:187–98. 10.2174/156800908784293659 18473732

[130] Fresno Vara JA, Casado E, de Castro J, Cejas P, Belda-Iniesta C, González-Barón M. PI3K/Akt signalling pathway and cancer. Cancer Treat Rev. 2004; 30:193–204. 10.1016/j.ctrv.2003.07.007 15023437

[131] Alzahrani AS. PI3K/Akt/mTOR inhibitors in cancer: At the bench and bedside. Semin Cancer Biol. 2019; 59:125–32. 10.1016/j.semcancer.2019.07.009 31323288

[132] Shi JJ, Xing H, Wang YX, Zhang X, Zhan QM, Geng MY, Ding J, Meng LH. PI3Kα inhibitors sensitize esophageal squamous cell carcinoma to radiation by abrogating survival signals in tumor cells and tumor microenvironment. Cancer Lett. 2019; 459:145–55. 10.1016/j.canlet.2019.05.040 31173854

[133] Song M, Liu X, Liu K, Zhao R, Huang H, Shi Y, Zhang M, Zhou S, Xie H, Chen H, Li Y, Zheng Y, Wu Q, et al. Targeting AKT with Oridonin Inhibits Growth of Esophageal Squamous Cell Carcinoma *In Vitro* and Patient-Derived Xenografts *In Vivo*. Mol Cancer Ther. 2018; 17:1540–53. 10.1158/1535-7163.MCT-17-0823 29695636 PMC6715294

[134] Liu X, Song M, Wang P, Zhao R, Chen H, Zhang M, Shi Y, Liu K, Liu F, Yang R, Li E, Bode AM, Dong Z, Lee MH. Targeted therapy of the AKT kinase inhibits esophageal squamous cell carcinoma growth *in vitro* and *in vivo*. Int J Cancer. 2019; 145:1007–19. 10.1002/ijc.32285 30887517 PMC6618024

[135] Liu F, Zu X, Xie X, Zhang Y, Liu K, Chen H, Wang T, Bode AM, Dong Z, Kim DJ. Scutellarin Suppresses Patient-Derived Xenograft Tumor Growth by Directly Targeting AKT in Esophageal Squamous Cell Carcinoma. Cancer Prev Res (Phila). 2019; 12:849–60. 10.1158/1940-6207.CAPR-19-0244 31554627

[136] Prager GW, Poettler M, Unseld M, Zielinski CC. Angiogenesis in cancer: Anti-VEGF escape mechanisms. Transl Lung Cancer Res. 2012; 1:14–25. 10.3978/j.issn.2218-6751.2011.11.02 25806151 PMC4367591

[137] Itatani Y, Kawada K, Yamamoto T, Sakai Y. Resistance to Anti-Angiogenic Therapy in Cancer-Alterations to Anti-VEGF Pathway. Int J Mol Sci. 2018; 19:1232. 10.3390/ijms19041232 29670046 PMC5979390

[138] Khan U, Shah MA. Ramucirumab for the treatment of gastric or gastro-esophageal junction cancer. Expert Opin Biol Ther. 2019; 19:1135–41. 10.1080/14712598.2019.1656715 31452409

[139] Wei B, Wang Y, Wang J, Cai X, Xu L, Wu J, Wang Y, Liu W, Gu Y, Guo W, Xu Q. Apatinib suppresses tumor progression and enhances cisplatin sensitivity in esophageal cancer via the Akt/β-catenin pathway. Cancer Cell Int. 2020; 20:198. 10.1186/s12935-020-01290-z 32514243 PMC7254695

[140] Fuchs CS, Tomasek J, Yong CJ, Dumitru F, Passalacqua R, Goswami C, Safran H, Dos Santos LV, Aprile G, Ferry DR, Melichar B, Tehfe M, Topuzov E, et al, and REGARD Trial Investigators. Ramucirumab monotherapy for previously treated advanced gastric or gastro-oesophageal junction adenocarcinoma (REGARD): an international, randomised, multicentre, placebo-controlled, phase 3 trial. Lancet. 2014; 383:31–39. 10.1016/S0140-6736(13)61719-5 24094768

[141] Wilke H, Muro K, Van Cutsem E, Oh SC, Bodoky G, Shimada Y, Hironaka S, Sugimoto N, Lipatov O, Kim TY, Cunningham D, Rougier P, Komatsu Y, et al, and RAINBOW Study Group. Ramucirumab plus paclitaxel versus placebo plus paclitaxel in patients with previously treated advanced gastric or gastro-oesophageal junction adenocarcinoma (RAINBOW): a double-blind, randomised phase 3 trial. Lancet Oncol. 2014; 15:1224–35. 10.1016/S1470-2045(14)70420-6 25240821

[142] Yanwei L, Feng H, Ren P, Yue J, Zhang W, Tang P, Shang X, Pang Q, Liu D, Chen C, Pan Z, Tao YZ. Safety and Efficacy of Apatinib Monotherapy for Unresectable, Metastatic Esophageal Cancer: A Single-Arm, Open-Label, Phase II Study. Oncologist. 2020; 25:e1464–72. 10.1634/theoncologist.2020-0310 32342599 PMC7543358

[143] Liang LJ, Wen YX, Xia YY, Wang L, Fei JY, Jiang XD. Apatinib combined with docetaxel as a salvage treatment for metastatic esophageal squamous cancer: a case report. Onco Targets Ther. 2018; 11:5821–26. 10.2147/OTT.S174429 30271164 PMC6145360

[144] Steins A, Ebbing EA, Pistorius MC, Waasdorp C, Krishnadath KK, Medema JP, Wilmink JW, Mathôt RA, Bijlsma MF, van Laarhoven HW. Systemic effects of angiogenesis inhibition alter pharmacokinetics and intratumoral delivery of nab-paclitaxel. Drug Deliv. 2017; 24:1801–10. 10.1080/10717544.2017.1406559 29172757 PMC8241153

[145] Slotta-Huspenina J, Becker KF, Feith M, Walch A, Langer R. Heat Shock Protein 90 (HSP90) and Her2 in Adenocarcinomas of the Esophagus. Cancers (Basel). 2014; 6:1382–93. 10.3390/cancers6031382 24978439 PMC4190546

[146] Yan H, Li B, Fan T, Jiang S, Wang R, Sun M. Clinical significance of serum dynamics of HSP90a level in esophageal squamous cell carcinoma patients treated with definitive chemoradiotherapy. Cancer Biomark. 2017; 19:185–92. 10.3233/CBM-160502 28387662 PMC13020712

[147] Ekman S, Bergqvist M, Tell R, Bergström S, Lennartsson J. Hsp90 as a therapeutic target in patients with oesophageal carcinoma. Expert Opin Ther Targets. 2010; 14:317–28. 10.1517/14728221003621278 20148718

[148] Guan L, Zou Q, Liu Q, Lin Y, Chen S. HSP90 Inhibitor Ganetespib (STA-9090) Inhibits Tumor Growth in c-Myc-Dependent Esophageal Squamous Cell Carcinoma. Onco Targets Ther. 2020; 13:2997–3011. 10.2147/OTT.S245813 32308431 PMC7156265

[149] Gao YB, Chen ZL, Li JG, Hu XD, Shi XJ, Sun ZM, Zhang F, Zhao ZR, Li ZT, Liu ZY, Zhao YD, Sun J, Zhou CC, et al. Genetic landscape of esophageal squamous cell carcinoma. Nat Genet. 2014; 46:1097–102. 10.1038/ng.3076 25151357

[150] Sawada G, Niida A, Uchi R, Hirata H, Shimamura T, Suzuki Y, Shiraishi Y, Chiba K, Imoto S, Takahashi Y, Iwaya T, Sudo T, Hayashi T, et al. Genomic Landscape of Esophageal Squamous Cell Carcinoma in a Japanese Population. Gastroenterology. 2016; 150:1171–82. 10.1053/j.gastro.2016.01.035 26873401

[151] Lubin DJ, Mick R, Shroff SG, Stashek K, Furth EE. The notch pathway is activated in neoplastic progression in esophageal squamous cell carcinoma. Hum Pathol. 2018; 72:66–70. 10.1016/j.humpath.2017.11.004 29137934

[152] Wang Z, Da Silva TG, Jin K, Han X, Ranganathan P, Zhu X, Sanchez-Mejias A, Bai F, Li B, Fei DL, Weaver K, Carpio RV, Moscowitz AE, et al. Notch signaling drives stemness and tumorigenicity of esophageal adenocarcinoma. Cancer Res. 2014; 74:6364–74. 10.1158/0008-5472.CAN-14-2051 25164006 PMC4527315

[153] Wang Z, Chen J, Capobianco AJ. The Notch signaling pathway in esophageal adenocarcinoma. Cell Mol Biol (Noisy-le-grand). 2015; 61:24–32. 26518893

[154] Jordan MA, Wilson L. Microtubules as a target for anticancer drugs. Nat Rev Cancer. 2004; 4:253–65. 10.1038/nrc1317 15057285

[155] Stanton RA, Gernert KM, Nettles JH, Aneja R. Drugs that target dynamic microtubules: a new molecular perspective. Med Res Rev. 2011; 31:443–81. 10.1002/med.20242 21381049 PMC3155728

[156] Sheng Y, Liu K, Wu Q, Oi N, Chen H, Reddy K, Jiang Y, Yao K, Li H, Li W, Zhang Y, Saleem M, Ma WY, et al. PPMP, a novel tubulin-depolymerizing agent against esophageal cancer in patient-derived tumor xenografts. Oncotarget. 2016; 7:30977–89. 10.18632/oncotarget.9050 27129160 PMC5058732

[157] Huarte M. The emerging role of lncRNAs in cancer. Nat Med. 2015; 21:1253–61. 10.1038/nm.3981 26540387

[158] Renganathan A, Felley-Bosco E. Long Noncoding RNAs in Cancer and Therapeutic Potential. Adv Exp Med Biol. 2017; 1008:199–222. 10.1007/978-981-10-5203-3_7 28815541

[159] Liu J, Liu ZX, Wu QN, Lu YX, Wong CW, Miao L, Wang Y, Wang Z, Jin Y, He MM, Ren C, Wang DS, Chen DL, et al. Long noncoding RNA AGPG regulates PFKFB3-mediated tumor glycolytic reprogramming. Nat Commun. 2020; 11:1507. 10.1038/s41467-020-15112-3 32198345 PMC7083971

[160] Ebbing EA, Steins A, Fessler E, Stathi P, Lesterhuis WJ, Krishnadath KK, Vermeulen L, Medema JP, Bijlsma MF, van Laarhoven HW. Esophageal Adenocarcinoma Cells and Xenograft Tumors Exposed to Erb-b2 Receptor Tyrosine Kinase 2 and 3 Inhibitors Activate Transforming Growth Factor Beta Signaling, Which Induces Epithelial to Mesenchymal Transition. Gastroenterology. 2017; 153:63–76.e14. 10.1053/j.gastro.2017.03.004 28286209

[161] Mashimo T, Takizawa A, Kobayashi J, Kunihiro Y, Yoshimi K, Ishida S, Tanabe K, Yanagi A, Tachibana A, Hirose J, Yomoda J, Morimoto S, Kuramoto T, et al. Generation and characterization of severe combined immunodeficiency rats. Cell Rep. 2012; 2:685–94. 10.1016/j.celrep.2012.08.009 22981234

[162] Miao J, Ying B, Li R, Tollefson AE, Spencer JF, Wold WS, Song SH, Kong IK, Toth K, Wang Y, Wang Z. Characterization of an N-Terminal Non-Core Domain of RAG1 Gene Disrupted Syrian Hamster Model Generated by CRISPR Cas9. Viruses. 2018; 10:243. 10.3390/v10050243 29734775 PMC5977236

[163] Miao JX, Wang JY, Li HZ, Guo HR, Dunmall LSC, Zhang ZX, Cheng ZG, Gao DL, Dong JZ, Wang ZD, Wang YH. Promising xenograft animal model recapitulating the features of human pancreatic cancer. World J Gastroenterol. 2020; 26:4802–16. 10.3748/wjg.v26.i32.4802 32921958 PMC7459204

[164] Lee NP, Chan CM, Tung LN, Wang HK, Law S. Tumor xenograft animal models for esophageal squamous cell carcinoma. J Biomed Sci. 2018; 25:66. 10.1186/s12929-018-0468-7 30157855 PMC6116446

